# Increased Sensitivity of the Circadian System to Temporal Changes in the Feeding Regime of Spontaneously Hypertensive Rats - A Potential Role for *Bmal2* in the Liver

**DOI:** 10.1371/journal.pone.0075690

**Published:** 2013-09-25

**Authors:** Lenka Polidarová, Martin Sládek, Marta Nováková, Daniela Parkanová, Alena Sumová

**Affiliations:** Department of Neurohumoral Regulations, Institute of Physiology, v.v.i., Academy of Science of the Czech Republic, Prague, Czech Republic; Karlsruhe Institute of Technology, Germany

## Abstract

The mammalian timekeeping system generates circadian oscillations that rhythmically drive various functions in the body, including metabolic processes. In the liver, circadian clocks may respond both to actual feeding conditions and to the metabolic state. The temporal restriction of food availability to improper times of day (restricted feeding, RF) leads to the development of food anticipatory activity (FAA) and resets the hepatic clock accordingly. The aim of this study was to assess this response in a rat strain exhibiting complex pathophysiological symptoms involving spontaneous hypertension, an abnormal metabolic state and changes in the circadian system, i.e., in spontaneously hypertensive rats (SHR). The results revealed that SHR were more sensitive to RF compared with control rats, developing earlier and more pronounced FAA. Whereas in control rats, the RF only redistributed the activity profiles into two bouts (one corresponding to FAA and the other corresponding to the dark phase), in SHR the RF completely phase-advanced the locomotor activity according to the time of food presentation. The higher behavioral sensitivity to RF was correlated with larger phase advances of the hepatic clock in response to RF in SHR. Moreover, in contrast to the controls, RF did not suppress the amplitude of the hepatic clock oscillation in SHR. In the colon, no significant differences in response to RF between the two rat strains were detected. The results suggested the possible involvement of the *Bmal2* gene in the higher sensitivity of the hepatic clock to RF in SHR because, in contrast to the Wistar rats, the rhythm of *Bmal2* expression was advanced similarly to that of *Bmal1* under RF. Altogether, the data demonstrate a higher behavioral and circadian responsiveness to RF in the rat strain with a cardiovascular and metabolic pathology and suggest a likely functional role for the *Bmal2* gene within the circadian clock.

## Introduction

Daily changes in activity and feeding are driven by an internal timekeeping system to occur synchronously at the same time during the day. The phasing of these rhythms is dependent on the species, being increased during the night in nocturnal species and during the day in diurnal species. The internal timekeeping system drives daily rhythms in feeding and activity even under conditions lacking daytime signals, such as constant darkness. Under these conditions, the rhythms exhibit approximately 24-hour periods and are thus called circadian. This mechanism not only drives the rhythms but also sets them to a proper phase in anticipation of upcoming changes in the external light/dark (LD) cycle. In mammals, rhythms in feeding and activity represent outputs of the so called "central" clock located in the hypothalamic area of the brain in the suprachiasmatic nuclei (SCN) [1,2]. The SCN are specialized to perceive information about external LD cycle due to their direct connection with the retina (for review, see [3]). The SCN clock adjusts its rhythmicity according to lighting information and passes the signal to so-called "peripheral" clocks, which are located in various bodily cells and drive rhythmically the tissue- and organ-specific programs (for review, see [4]). As a result, the processes driven by the central and peripheral clocks are synchronized. At the cellular level, the molecular mechanism responsible for generating circadian oscillations is composed of interconnected transcriptional/translational feedback loops among the clock genes and their protein products (for review, see [5]). The core clockwork consists of the transcriptional activators CLOCK and BMAL1, which bind as a heterodimer to E-box elements in the promoters of the clock genes *Per1*, *Per2*, *Cry1*, *Cry2*, *Rev-erbα/β* and *Rorα/β/γ* and drive their transcription. The PER1,2 and CRY1,2 proteins accumulate in the cytoplasm, form complexes and translocate into the nucleus, where they inhibit the transcriptional activity of CLOCK-BMAL1. This feedback mechanism results in the rhythmic expression of the clock genes. Furthermore, REV-ERBα and RORα create accessory feedback loops by binding to the promoter in *Bmal1* to repress or activate its transcription, respectively. *Bmal2* is a paralog of *Bmal1* [6], and its expression from a constitutively expressed promoter can restore the clock and metabolic phenotypes of *Bmal1*-knockout mice [7]. The RORα-mediated activation of *Bmal1* transcription is enhanced by the PPARγ coactivator 1α (PGC1α) [8]. Members of the PAR bZIP transcription factor family, including the activator DBP and the repressor E4BP4 [9,10], act via D-box elements in their target genes to form additional accessory feedback loops. Posttranslational modifications, such as phosphorylation, ubiquitination, sumoylation, and acetylation/deacetylation, enhance the robustness and stability of the clock mechanism (for review, see [11]). The rhythmic regulation of E-box elements by the BMAL1/CLOCK complex, D-box elements by PAR bZIP factors, or RORE sequences by RORα/REV-ERBα, drive the rhythmic expression of so-called clock-controlled genes, coding transcriptional factors or functional proteins involved in the regulation of various physiological pathways [12].

In nature, food is rarely present *ad libitum*, and feeding regimes are affected by many factors. Under laboratory conditions, limiting food access to a certain time of day, so-called restricted feeding (RF), leads to the development of food-anticipatory behavior in advance of food availability, including increases in locomotor activity, body temperature [13], corticosterone levels [14], and plasma levels of ketone body and free fatty acids [15]. Food-anticipatory activity (FAA) is driven independently of the central clock because it occurs in SCN-lesioned animals ( [16], reviewed by [17]) as well as in animals with a genetically ablated clock [18,19]. This phenomenon is thought to be driven by another oscillator, the so called food-entrainable clock, as it persists in conditions without periodic food availability [20]. The food-entrainable oscillator likely communicates with the circadian clock, as FAA is absent when the period of the feeding cycle is too distant from 24 h [21,22,23]. Moreover, this SCN-independent oscillator may depend partially on the canonical circadian clock components, as deficiencies in individual clock genes lead to variations in the circadian range of FAA entrainment [24]. Therefore, clock genes are likely involved in the regulation of FAA in a circadian oscillatory manner. However, in spite of significant efforts to determine its localization in the body, the oscillator has not yet been identified (for review, see [17,25]).

The SCN pathways entraining the peripheral clocks are of multiple origin (for review, see [4]). The feeding rhythm is one of the signals that may provide temporal information to the periphery, as many peripheral clocks, including the clocks in the pancreas, kidney, heart, adipose tissue, liver and intestine, adjust their phases according to temporal changes in food availability, whereas the SCN clock remains unaffected, always running in phase with the LD cycle [26,27,28,29,30]. Thus, under RF conditions, the timing of food availability dominates the signals in the peripheral organs about the external LD cycle from the SCN. However, the mechanism of peripheral clock entrainment by the feeding regime is not yet fully understood.

It seems that the higher sensitivity of the peripheral clocks to the signals arising from the periodic food availability compared to the signals from the central clock in the SCN is the result of an evolutionary strategy to accelerate adjustment of the peripheral clocks located in metabolic tissues to abrupt and unpredictable changes in food availability, irrespective of the external LD regime. It is therefore plausible to speculate that the sensitivity to RF might differ among animal species and be affected in situations when metabolic functions are disordered. The spontaneously hypertensive rat (SHR) has recently been described as a rat strain with an aberrant circadian system [31]. These changes correlate with previous findings that the strain is not only hypertensive but also predisposed to metabolic diseases [32]. For example, the hepatic steatosis, i.e., "fatty liver", phenotype in the SHR was associated with the transcription factor Srebp1 (sterol regulatory element factor binding protein 1), which regulates hepatic cholesterol levels and influences the susceptibility to dietary-induced accumulation of liver cholesterol [33]. A direct connection between this pathology and the circadian clock function in SHR has not been recognized. However, several polymorphisms associated with metabolic syndrome were identified in the SHR *Bmal1* promoter, suggesting a potential link between the circadian system and the SHR pathological phenotype [34]. In SHR, a regular feeding regime affected the hypertensive phenotype by restoring a diurnal rhythm in the blood pressure as well as clock- and metabolism-related gene expression in cardiovascular tissues [35]. In addition, caloric restriction prevented hypertension in SHR [36]. However, the sensitivity of SHR to metabolic challenge, such as temporal restriction of food availability to an improper time of a day, has not been examined. There is a possibility that due to the altered circadian system [31], SHR may respond differently to changes in feeding regime. To test this hypothesis, the main goal of the current study was to compare the sensitivity of the circadian system to a RF regime between the SHR and a normotensive control rat strain without any metabolic pathology. The comparison was performed at the behavioral level as well as at the level of the molecular clockwork in the liver and colon. To increase the urgency of the signal, food was provided only during the daytime, i.e., at an improper time for a nocturnal animal. The results demonstrate significant differences in the sensitivity of the circadian system of the SHR to the metabolic challenge compared with controls at both the behavioral and gene expression level.

## Methods

### Ethics statement

All experiments were approved by the Animal Care and Use Committee of the Institute of Physiology in agreement with the Animal Protection Law of the Czech Republic as well as the European Community Council directives 86/609/EEC. All efforts were made to minimize the suffering of the animals.

### Experimental animals

Two-month-old male SHR/Ola (Institute of Physiology, Academy of Sciences of the Czech Republic) and Wistar:Han rats (Velaz s.r.o., Czech Republic) were maintained at least 4 weeks at a temperature of 21 ± 2°C in an alternating LD regime with 12 h of light and 12 h of darkness per day with free access to food and water. The lights were turned on at 06:00 h and off at 18: 00 h. The time at which the lights were turned on was designated as Zeitgeber time (ZT) 0, and the time at which the lights were turned off was designated as ZT12. Light was provided by overhead 40-W fluorescent tubes, and illumination was between 50 and 300 lux, depending on the cage position in the animal room.

### Experimental protocol

Rats of both strains were maintained under LD12: 12 with free access to food and water for 16 days. During the next 10 days in LD12: 12, the animals were subjected to an RF protocol, i.e., the rats were provided food for only 6 h during the daytime (between ZT3 and ZT9). Access to drinking water was not limited. On the day of sampling, the animals were released into constant darkness. The rats were monitored for spontaneous activity during the entire 26-day period leading up to the sampling.

To determine the daily gene expression profiles, animals of both strains were sampled during the first day in constant darkness, starting at the time of the previous lights on, which was designed as circadian time 0 (CT0), and continuing every 4 h throughout the 24-h circadian cycle. Three to five rats per time point were killed by decapitation under deep anesthesia, which occurred after approximately 1-2 min (i.p. injection of thiopental, 50 mg per kg), and samples of the brain, liver and colon were collected.

### Locomotor activity and feeding monitoring

To monitor locomotor activity, rats of both strains were maintained individually in cages equipped with infrared movement detectors that were attached above the center of the cage top. A circadian activity monitoring system (Dr. H.M. Cooper, INSERM, France) was used to measure activity every minute, and double-plotted actograms were generated to evaluate the activity. The resulting data, including calculations of activity before and after food presence, were analyzed using ClockLab toolbox (Actimetrics, Illinois, USA).

For the analysis of spontaneous feeding behavior, 3 SHR fed *ad libitum* were maintained individually in cages and were monitored with infrared-sensitive video cameras attached above the cage top. The records were analyzed for three consecutive days. The time the rats spent eating pellets during the day and night was calculated for each animal, data were cumulated for mean daytime and nighttime eating and expressed as the means ± S.E.M. of the percent of the total daily time spent eating.

To determine the impact of RF on body weight gain and amount of food consumed, 5 rats of each strain were weighted before, and then 5 days and 10 days after the beginning of RF. Also, food consumption was monitored by weighting the pellets before and after they were provided to rats every day of the RF.

### Tissue sampling

Immediately following their removal, the brains were frozen on dry ice and kept at -80°C. The brains were sectioned throughout the entire rostro-caudal extent of the SCN into five series of 12 µm thick slices in an alternating order. The sections were further processed for *in situ* hybridization to determine the gene expression profiles in the SCN.

Dissected samples of the liver were immersed in RNAlater stabilization reagent (Qiagen, Valencia, USA). The distal part of the colon was cut just above the pelvic brim, the samples were rinsed with phosphate-buffered saline and cut longitudinally. The mucosal layer was gently scraped, yielding material rich in epithelial cells that was subsequently immersed in RNAlater stabilization reagent. All samples were stored at 4°C for no longer than 1 week prior to isolation of the total RNA and subsequent real-time qRT-PCR.

### In situ hybridization

The cDNA fragments of rat *rPer2* (1512 bp; 369-1881; GenBank NM_031678), *rRev-erbα* (1109 bp; 558-1666; GenBank BC062047) and *rBmal1* (841 bp; 257-1098; GenBank AB012600) were used as templates for the *in vitro* transcription of cRNA probes. The probes were labeled using ^35^S-UTP, and the *in situ* hybridizations were performed as previously described [37]. The brain sections were hybridized for 20 h at 60°C. Following a post-hybridization wash, the sections were dehydrated in ethanol and dried. Finally, the slides were exposed to BIOMAX MR film (Kodak, USA) for 10-14 days and developed using the ADEFO-MIX-S developer and ADEFOFIX fixer (ADEFO-CHEMIE Gmbh, Germany). Brain sections were processed simultaneously under identical conditions. Autoradiographs of the sections were analyzed using an image analysis system (Image Pro, Olympus, New York, USA) to detect the relative optical density (OD) of the specific hybridization signal in the area of the SCN.

### RNA isolation and Real-time qRT-PCR

Total RNA was extracted by homogenization from the liver and by sonication from the colon and subsequently purified using the RNeasy Mini kit (Qiagen, Valencia, USA) according to the manufacturer’s instructions. RNA concentrations were determined by spectrophotometry at 260 nm, and the RNA quality was assessed by electrophoresis on a 1.5% agarose gel. Moreover, the integrity of randomly selected samples of total RNA was tested using an Agilent 2100 Bioanalyzer (Agilent Technologies, Santa Clara, USA).

The qRT-PCR method used to detect the clock genes has been described previously [38]. Briefly, 1 µg of total RNA was reverse transcribed using the SuperScript VILO cDNA synthesis kit (Invitrogen, Carlsbad, USA) with random primers. The resulting cDNAs were used as templates for qRT-PCR. Diluted cDNA was amplified on a LightCycler 480 (Roche, Basel, Switzerland) using the Express SYBR GreenER qPCR SuperMix (Invitrogen, Carlsbad, USA) and the corresponding primers (sequences were published previously [31] with the exception of the Prkab2 primers: forward 5'-TGA GCC TCA CCT TAG AGC CT-3', reverse 5'-AGG ACC AAC CGA GAC ACA AC-3'). Relative quantification was achieved using a standard curve and subsequently normalizing the gene expression to *β2-microglobulin* (B2M), which has been used as a housekeeping gene previously [38]. Its expression was stable throughout the day and did not vary between the analyzed tissues.

To compare the differences between the two rat strains (SHR versus Wistar rats) and the two experimental conditions (*ad libitum* feeding and RF) in the amplitudes, mesors and acrophases of the gene expression profiles, the samples of each rat strain under feeding *ad libitum* and RF, as well as of both strains under RF, were analyzed in the same run of qRT-PCR.

### Western blotting of BMAL2

5 Wistar rats and 5 SHR were kept on RF for 10 days and then released in DD. 1 Wistar rat and 1 SHR were killed every 6 h during the 24 h cycle. Samples of liver were removed, frozen on dry ice and homogenized in 1ml of CellLytic MT extraction reagent (Sigma, USA) with 1x Protease inhibitor cocktail (Sigma, USA) using SilentCrusher S (Heidolph, Germany). Protein concentration was determined by Bradford assay (Thermo Scientific, USA). All reagents for Western blot were purchased from Life technologies, USA, unless stated otherwise. Approximately 25 µg of total protein was mixed with NuPAGE LDS Sample buffer and Sample reducing reagent, denatured at 70°C for 10 min and separated with protein ladder on NuPAGE Bis-Tris 4-12% premade gel using NuPAGE MOPS SDS running buffer with antioxidant according to manufacturer’s instruction. The protein was transferred by electroblotting in NuPAGE transfer buffer with 10% methanol onto nitrocellulose membrane according to manufacturer’s instruction. The membrane was blocked in StartingBlock T20 blocking buffer (Sigma, USA) for 30 min and then incubated with primary antibody against BMAL2 (cat. no. SAB2100154, Sigma, USA) diluted 1:1000 in blocking buffer overnight at 4°C on a rocker. The membrane was then washed 5 times for 5 min in TBST (2.42 g/l Tris-HCl, 8 g/l NaCl, 0.1% Tween 20, pH 7.6) and incubated with secondary anti-rabbit HRP-conjugated antibody (Promega, USA) diluted 1:20000 in blocking buffer at room temperature (RT) for 1 h on a rocker. The membrane was then washed 5 x in TBST, incubated with SuperSignal West Femto Chemiluminescent substrate (Pierce, USA) and immunoreactive bands were detected after 5 min exposition using cooled camera system. The membrane was subsequently incubated for 30 min at RT in stripping buffer (62.5 mM Tris-HCl, 20 g/l SDS, 0.7 mM 2-Mercaptoethanol), washed 5 x in TBST, blocked in blocking buffer for 30 min, incubated with anti-β-actin HRP-conjugated monoclonal antibody (Sigma, USA) 1:20000 in blocking buffer for 1 h at RT, washed 5 x in TBST, incubated with West Pico substrate (Pierce, USA) and exposed for 12 s. Photographs of blots were imported into ImageJ (NIH, USA) software, where optical density of immunoreactive bands corresponding to the predicted protein size of BMAL2 was quantified relative to β-actin internal standard.

### Statistical analysis

The differences in locomotor activity (i.e., the values of the total 24 h-activity, the activity determined during 3-h-time intervals and the food anticipatory activity), body weight and food consumption during RF between SHR and Wistar rats were evaluated by two-way ANOVA followed by Bonferroni multiple comparisons test with P < 0.05 required for significance. The weight gains of Wistar rats and SHR during RF were evaluated by Student’s *t* test with P < 0.05 required for significance.

For the gene expression profiles, data were fit to two alternative regression models to differentiate between rhythmic and non-rhythmic expression: either a horizontal straight line (null hypothesis) or a single cosine curve (alternative hypothesis), defined by the equation Y=mesor+(amplitude*cos(2*π* (X-acrophase)/wavelength)) with a constant wavelength of 24 h. The extra sum-of-squares F test was used for comparison, and the cosine curve parameters were calculated unless the P value exceeded 0.05. The amplitude (i.e., the difference between the peak or trough and the mean value of a cosine curve), acrophase (i.e., the phase angle of the peak of a cosine curve), mesor (i.e., the average value around which the variable oscillates) and coefficient of determination R^2^ (i.e., goodness of fit) were compared where applicable. The least-squares regression method implemented in the Prism 5 software (GraphPad, La Jolla, USA) was applied. The differences in acrophases, amplitudes and mesors between the profiles in the SHR and Wistar rats were evaluated by Student’s *t* test and where applicable, the test was corrected for multiple comparisons. *The results of the Student’s t test are expressed as t*_*x*_ *values (with x in the lower index representing degrees of freedom*)* and as P value (level of significance*)*, with P < 0.05 required for significance*.

## Results

### Effect of RF on the circadian rhythm in the behavioral activity in SHR and Wistar rats

The recording of the feeding behavior of the SHR (n = 3) revealed that under feeding *ad libitum*, 34.9 ± 6.9% of their total feeding time occurred during the daytime and 65.1 ± 6.9% during the nighttime.

**Figure 1:**
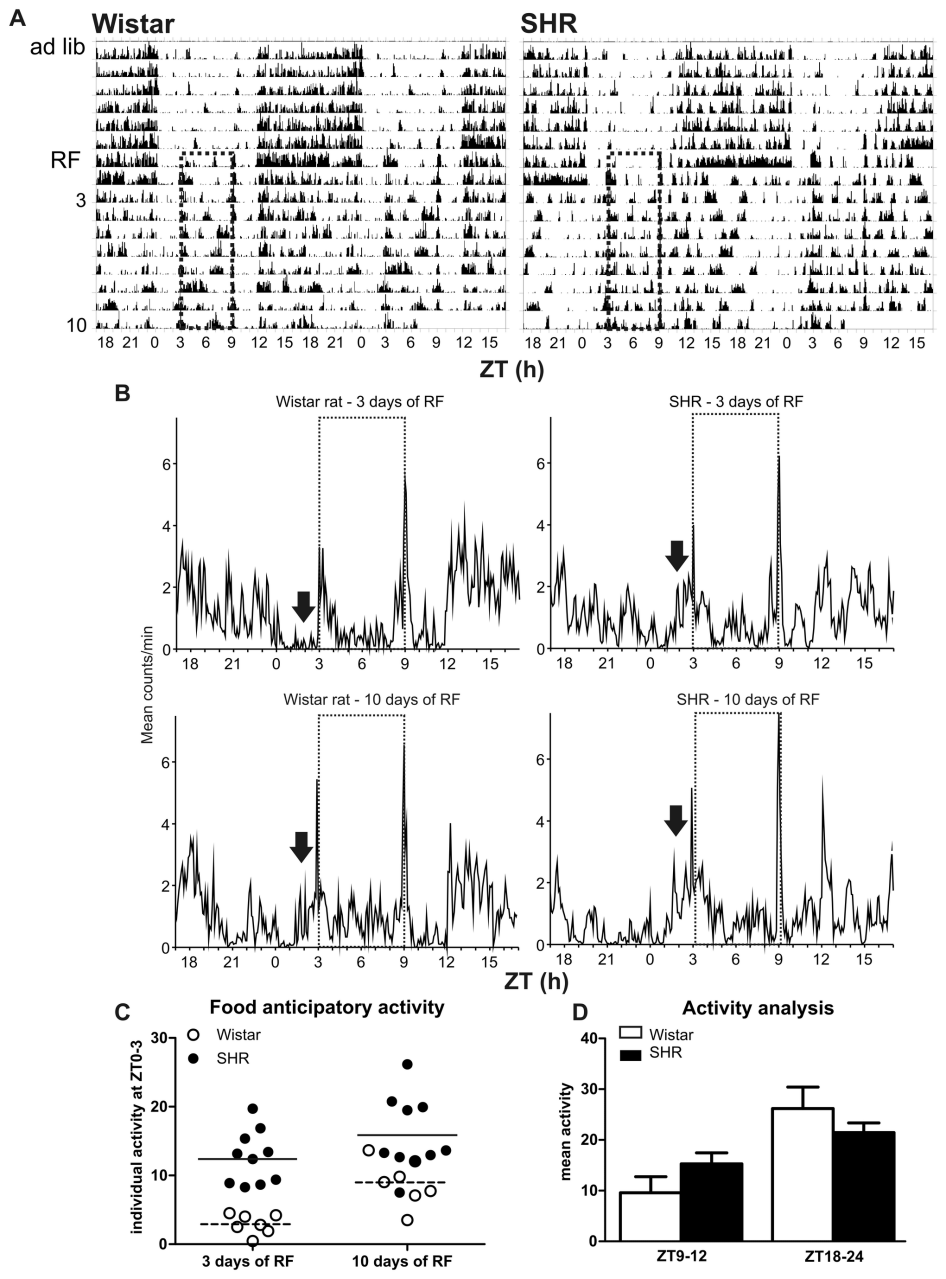
Analysis of the behavioral activity of Wistar rats and SHR maintained under restricted feeding conditions. **A**) **Representative** **double-plotted** **actograms** of the locomotor activity of a Wistar rat (left) and SHR (right) maintained under LD12: 12. The rats were initially fed *ad* *libitum* (ad lib), and thereafter, food was provided only during a 6-h period between Zeitgeber time (ZT) 3 and ZT9 for 10 days; the 6-h period of restricted feeding (RF) is depicted by the dashed rectangles. **B**) **Cumulative** **daily** **locomotor** **activity** **profiles** of Wistar rats (left, n = 7) and SHR (right, n = 10) detected on the 3rd day (upper panel) and on the 10th day (lower panel) of RF. The dotted rectangles represent the time of food availability; the arrows point at food anticipatory activity. **C**) **Comparison** **of** **food** **anticipatory** **activity** measured during the 3-h interval prior to food presentation, i.e., between ZT0 and ZT3, in individual Wistar rats (open circles) and SHR (full circles), expressed as the percent of total activity of each animal. **D**) **Comparison** **of** **activity** **level** (expressed as means ± S.E.M. in percent of total activity) in Wistar rats (open column; n = 7) and SHR (full column; n = 10) during the intervals between ZT9 and ZT12, i.e., between the end of the food presentation and the beginning of the dark period, and between ZT18 and ZT24, i.e., during the second half of the dark period.

Locomotor activity was recorded in the SHR (n = 10) and Wistar rats (n = 7) maintained under LD12: 12 and fed *ad libitum* prior to subjection to RF. The representative actograms of one Wistar rat and one SHR are shown in Figure 1A. Under conditions of feeding *ad libitum*, the total activity in counts per day (mean ± S.E.M.) was 4039 ± 846 in the SHR and 3594 ± 1426 in the Wistar rats, and under conditions of RF, the total activity was 3830 ± 866 in the SHR and 3782 ± 1258 in the Wistar rats. The two-way ANOVA did not reveal statistically significant differences in total activity levels between strains (P = 0.820) and feeding conditions (P = 0.992). Therefore, the overall activity was not affected by the RF regime in either strain.

RF affected the distribution of activity during the 24 h period: the rats became active during the time of food presence and also during a certain interval prior to feeding, i.e., they exhibited FAA (Figure 1A and 1B). Two-way ANOVA revealed that the FAA, measured as the mean activity between ZT0 and ZT3 throughout the duration of the RF regime, was significantly higher in the SHR than in the Wistar rats (P < 0.001). The analysis of the mean activity profiles of all SHR and Wistar rats (Figure 1B) revealed that FAA appeared on the 3^rd^ day of RF in SHR but only later in Wistar rats. In Wistar rats, the FAA on the 3^rd^ day of RF was significantly lower than that on the 10^th^ day of RF (P < 0.05), whereas in the SHR, the food anticipatory activity on the 3^rd^ day already reached the same level as on the 10^th^ day of RF (P > 0.05). Moreover, the amount of FAA was quantified as the % of the total activity in individual rats of both strains during the 3 h interval prior to food presence, i.e., between ZT0 and ZT3, on the 3^rd^ and 10^th^ day of RF (Figure 1C). The data revealed that the FAA was significantly more pronounced in SHR compared with Wistar rats on the 3rd (P < 0.0001) as well as on the 10th (P < 0.01) day of RF. Therefore, the data demonstrate that SHR develop FAA much earlier than Wistar rats and that the FAA is higher in SHR compared with Wistar rats.

A detailed inspection of the actograms from rats maintained under LD12: 12 and subjected to RF revealed that the activity of Wistar rats during the day was split into two intervals, one corresponding to the time of food presence and the short period prior, and the other corresponding to the dark phase of the LD cycle, i.e., starting at ZT12 and ending at approximately ZT24. Both intervals were interrupted with a 3-h interval of inactivity (ZT9 - ZT12). In contrast, the SHR became active prior to the food presence and remained active throughout the rest of the day and for most of the night, often ending their activity well before lights-on (Figure 1A). The analysis of activity during the intervals between ZT9 and ZT12 and between ZT18 and ZT24 over the entire period of RF (Figure 1D) by two-way ANOVA with Bonferroni multiple comparisons test revealed that compared with the Wistar rats, the SHR were significantly more active during the interval between the end of food presence and lights-off (P < 0.001) and less active during the 6-h interval prior to lights-on (P < 0.01). Therefore, in the SHR exposure to RF shifted the nocturnal activity to the time of food presence, whereas it did not affect the timing of nocturnal activity in the Wistar rats.

Effect of RF on body weight and food consumption was measured in SHR (n = 5) and Wistar rats (n = 5) (Figure S1). SHR exhibited a lean phenotype because in spite of the same age, the SHR body weight was lower compared with that of Wistar rats. During the 10 days of RF, body weight slightly increased in both rat strains (P < 0.05 for Wistar rats, P < 0.01 for SHR) (Figure S1A). There was no significant difference in the absolute increase in body weight between both strains (Figure S1C; P = 0.520), but SHR gained significantly more relative to their body weight (Figure S1D; P < 0.05). Recording of food consumption of individual rats each day on the RF (Figure S1B) revealed that SHR consumed slightly less food in the absolute amount (P < 0.05), but much more relative to their body weight (P < 0.001) compared with Wistar rats.

### Effect of RF on the circadian rhythm in the clock gene expression in the SCN of SHR

**Figure 2:**
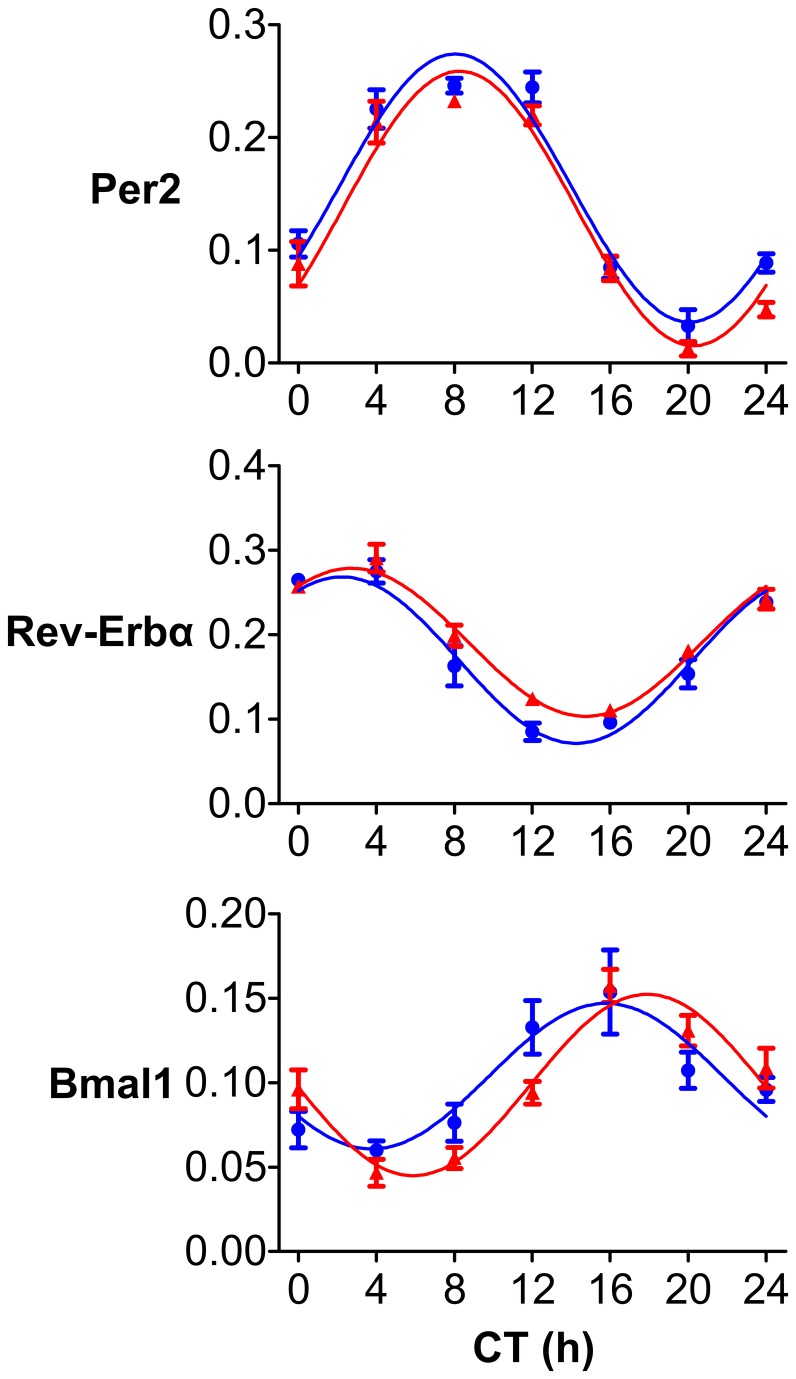
Effect of restricted feeding regime on daily clock gene expression profiles in the suprachiasmatic nuclei of SHR. SHR were maintained under LD12: 12 and fed *ad* *libitum* (blue circles and blue line) or exposed to a restricted feeding regime for 10 days (red triangles and red line). Thereafter, rats of both groups were released into constant darkness and sampled every 4 h during the next 24-h interval. The Levels of *Per2*, *Rev-erbα* and *Bmal1* mRNA were detected by *in* *situ* hybridization in the middle section of the SCN. Data are expressed as relative optical density, and each point represents the mean ± S.E.M. of 3 animals. Time is expressed as circadian time (CT), with CT0 corresponding to lights-on in the previous LD cycle. Data were fitted by cosine curves to calculate the acrophases, amplitudes and mesors (for details, see Methods).

Daily profiles of *Per2*, *Rev-erbα* and *Bmal1* expression were detected in the SCN of SHR maintained under LD12: 12 and fed *ad libitum* or subjected to RF (Figure 2). The expression profiles of all of the studied clock genes exhibited significant circadian rhythms (P < 0.001) and did not differ in their amplitudes or mesors under either experimental condition. The acrophases (mean ± S.E.M.) of the *Per2* and *Rev-erbα* expression profiles in rats fed *ad libitum* (8.05 ± 0.25 and 2.23 ± 0.29, respectively) and in those exposed to RF (8.27 ± 0.25 and 2.72 ± 0.27, respectively) were not significantly different (t_38_ = -0.619, P = 0.539 and t_38_ = -1.228, P = 0.227). For *Bmal1* expression, a delay in acrophase of the profile under RF (15.78 ± 0.63) compared with that under *ad libitum* feeding (17.90 ± 0.35) was detected (t_39_ = -2.914, P = 0.006). The results of cosinor analysis of the clock gene expression profiles in the SCN of SHR are summarized in Table S1.

To compare the clock gene expression profiles in the SCN of SHR and Wistar rats maintained under RF, *Per2* and *Bmal1* mRNA levels were expressed as the % of the maximal levels for each strain (data not shown), and the acrophases of the profiles were detected by cosinor analysis. No differences between the phases of the *Per2* and *Bmal1* expression profiles in the SCN of Wistar rats and SHR maintained under RF were found.

### Effect of RF on the circadian rhythm in the clock gene expression in the liver and colon of SHR and Wistar rats

In order to compare the daily profiles of clock gene expression in the liver and colon of each rat strain under *ad libitum* feeding conditions and RF, these profiles were assessed in the same run of qRT-PCR. The results of cosinor analysis of the clock gene expression profiles in the liver and colon of SHR and Wistar rats are summarized in Tables S2 and S3, respectively.

#### Expression profiles in the liver

**Figure 3:**
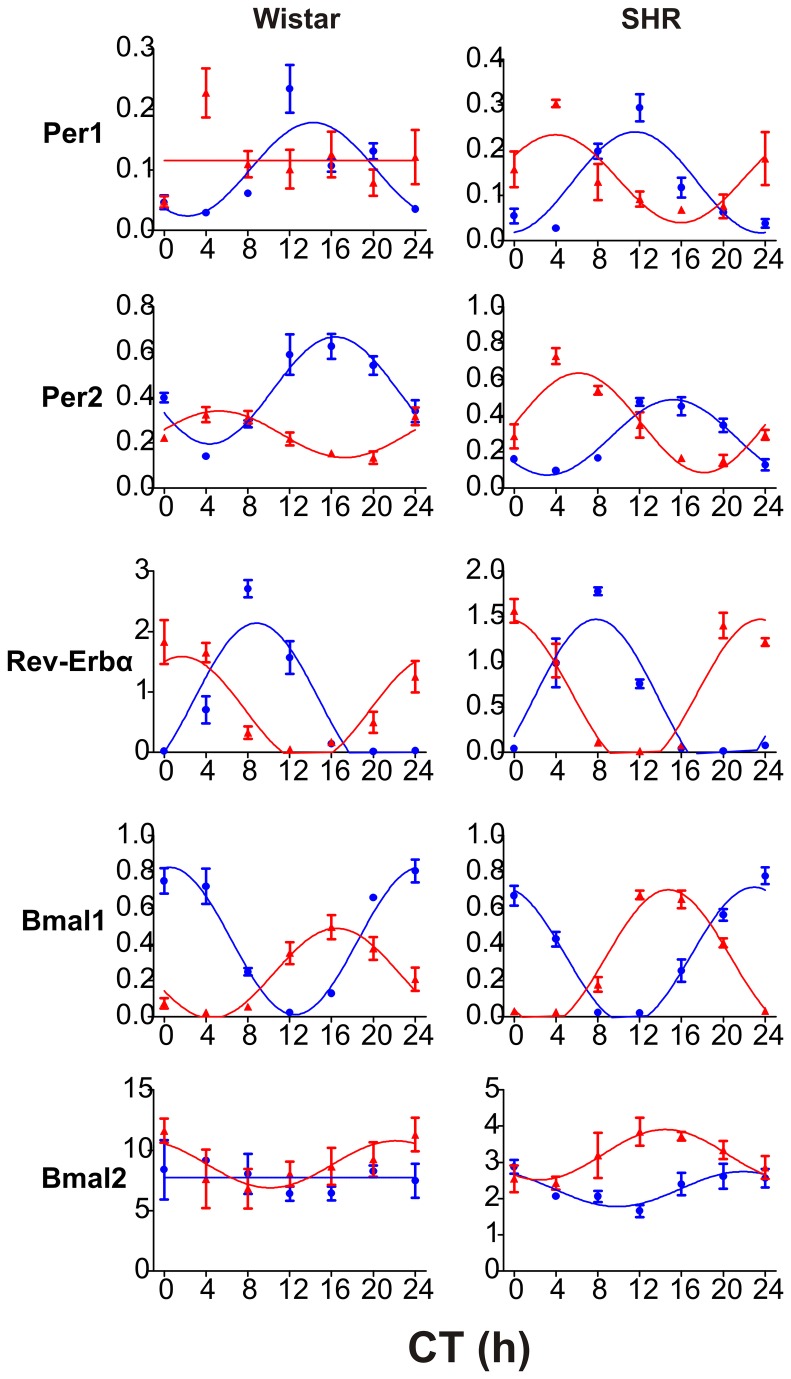
Effect of restricted feeding regime on daily profiles of clock gene expression in the liver of Wistar rats and SHR. The Wistar rats (left column) and SHR (right column) were maintained under LD12: 12 and fed *ad* *libitum* (blue circles and blue line) or exposed to a restricted feeding regime for 10 days (red triangles and red line). Daily expression profiles of the clock genes *Per1*, *Per2*, *Rev-erbα*, *Bmal1* and *Bmal2* were detected by qRT- PCR in the liver. Data are expressed as relative expression, and each point represents the mean ± S.E.M. of 3 (SHR) or 5 (Wistar rat) animals. Time is expressed as circadian time (CT), with CT0 corresponding to lights-on in the previous LD cycle. Data were fitted by cosine curves to calculate the acrophases, amplitudes and mesors (for details, see Methods).

As expected, the exposure to RF phase-advanced the clock gene expression profiles in the livers of the Wistar rats (Figure 3); the acrophases of *Per2*, *Rev-erbα* and *Bmal1* were significantly advanced in RF compared with *ad libitum* feeding (*Per2*: t_52_ = 14.111, P < 0.001; *Rev-erbα*: t_52_ = 9.208, P < 0.001; *Bmal1*: t_52_ = -28.166, P < 0.001). The expression profiles of *Per1* under RF and *Bmal2* under *ad libitum* conditions did not exhibit circadian variation. Importantly, in the Wistar rats, the expression profiles under RF conditions were significantly suppressed compared with *ad libitum* feeding in all studied clock genes (with the exception of *Bmal2*), because their amplitudes were significantly lower (*Per2*: t_52_ = 2.557, P = 0.014; *Rev-erbα*: t_52_ = 2.166, P = 0.035); *Bmal1*: t_52_ = 3.709, P < 0.001, and the *Per1* expression profile on RF was not rhythmic at all. Moreover, in the *Per2* and *Bmal1* expression profiles, not only the amplitudes but also the mesors were decreased in RF compared with *ad libitum* feeding (Per2: t_52_ = 9.366, P < 0.001; *Bmal1*: t_52_ = 5.974, P < 0.001). Interestingly, whereas under RF, the circadian expression of *Per1*, *Per2*, *Rev-erbα* and *Bmal1* was suppressed, *Bmal2* was expressed rhythmically. The acrophase of the *Bmal2* expression profile was delayed by approximately 5 to 6 h compared with the acrophase of the *Bmal1* profile under RF conditions and was very close to the phase of the *Bmal1* profile under *ad libitum* feeding conditions.

Similar to the Wistar rats, the clock gene expression profiles in the livers of the SHR were also phase advanced under RF compared with *ad libitum* feeding (*Per1*: t_38_ = 7.935, P < 0.001; *Per2*: t_38_ = 17.488, P < 0.001; *Rev-erbα*: t_38_ = -29.389, P < 0.001; *Bmal1*: t_38_ = 24.388, P < 0.001). In contrast to the Wistar rats, the *Bmal2* expression profile under *ad libitum* feeding was expressed rhythmically, and under RF the acrophase was significantly phase-advanced compared with *ad libitum* feeding (t_38_ = 5.242, P < 0.001). Moreover, in the SHR the acrophases of *Bmal1* and *Bmal2* expression profiles were approximately the same, and the synch was maintained under both *ad libitum* and RF conditions. Again, in contrast to the Wistar rats, the rhythmic expression under RF was either not suppressed in *Per1*, *Rev-erbα*, *Bmal1*, or even upregulated in *Per2* (mesor t_38_ = -3.135, P = 0.003) and *Bmal2* (mesor t_38_ = -6.237, P < 0.001) compared with *ad libitum* feeding.

The data demonstrate that whereas the RF regime suppresses the rhythmicity of the hepatic clock in Wistar rats, it enhances its rhythmicity in SHR.

#### Expression profiles in the colon

The results of the cosinor analysis of the clock gene expression profiles in the colons of SHR and Wistar rats are summarized in Table S3.

**Figure 4:**
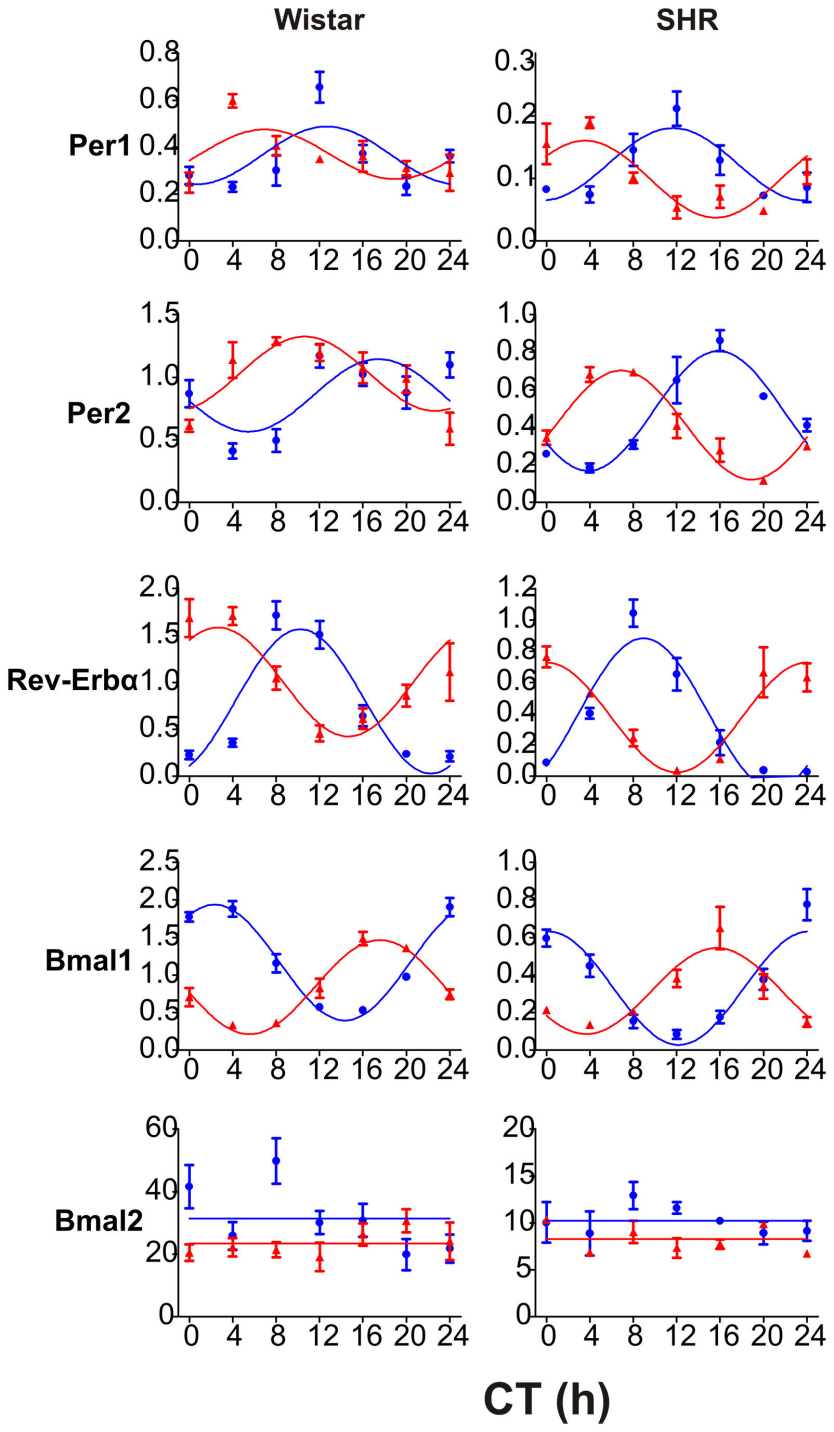
Effect of a restricted feeding regime on the daily profiles of clock gene expression in the colon of Wistar rats and SHR. The Wistar rats (left column) and SHR (right column) were maintained under LD12: 12 and fed *ad* *libitum* (blue circles and blue line) or exposed to a restricted feeding regime for 10 days (red triangles and red line). Daily expression profiles of the clock genes *Per1*, *Per2*, *Rev-erbα*, *Bmal1* and *Bmal2* were detected by qRT- PCR in the colon. For other details see Figure 3.

Again, as expected, the exposure of Wistar rats to RF phase-advanced the clock gene expression profiles in the colon (Figure 4); the acrophases of the rhythmically expressed genes, i.e. *Per1, Per2*, *Rev-erbα* and *Bmal1*, were significantly advanced in RF compared with *ad libitum* feeding (*Per1*: t_62_ = 3.554, P < 0.001; *Per2*: t_62_ = 5.702, P < 0.001; *Rev-erbα*: t_62_ = 10,264, P < 0.001; *Bmal1*: t_62_ = -43,710, P < 0.001). The expression profiles of *Bmal2* in the colon did not exhibit circadian variation under *ad libitum* nor RF conditions and did not differ under either condition. A decline in the amplitude of the rhythms was suggested in *Per2* and *Rev-erbα* and was statistically significant for *Bmal1* (t_66_ = 2.183, P = 0.033). Therefore, the rhythmicity of the colonic clock was either not affected or rather suppressed under RF compared with *ad libitum* conditions.

In the SHR, the RF conditions phase-advanced the colonic clock similarly to that in the Wistar rats, with the acrophases of *Per1, Per2*, *Rev-erbα* and *Bmal1* significantly advanced in RF compared with *ad libitum* feeding (*Per1*: t_38_ = 7.346, P < 0.001; *Per2*: t_38_ = 18.208, P < 0.001; *Rev-erbα*: t_38_ = 14.456, P < 0.001; *Bmal1*: t_38_ = -21.725, P < 0.001). The *Bmal2* expression did not exhibit circadian variation and did not differ between the RF and *ad libitum* feeding conditions. The amplitudes of the *Rev-erbα* and *Bmal1* expression profiles were significantly decreased (*Rev-erbα*: t_38_ = 2.136, P = 0.039 and t_38_ = 2.183, P = 0.033), and the mesor of the *Per2* expression profile was significantly lower (t_38_ = 2.562, P = 0.014) under RF conditions compared with *ad libitum* feeding. Therefore, the colonic clock of the SHR was significantly suppressed under RF compared with *ad libitum* feeding.

The data demonstrate that in contrast to the liver, RF suppresses the rhythmicity of the colonic clock in both rat strains.

### Comparison of the responsiveness of the hepatic and colonic clocks to RF in SHR and Wistar rats

**Figure 5:**
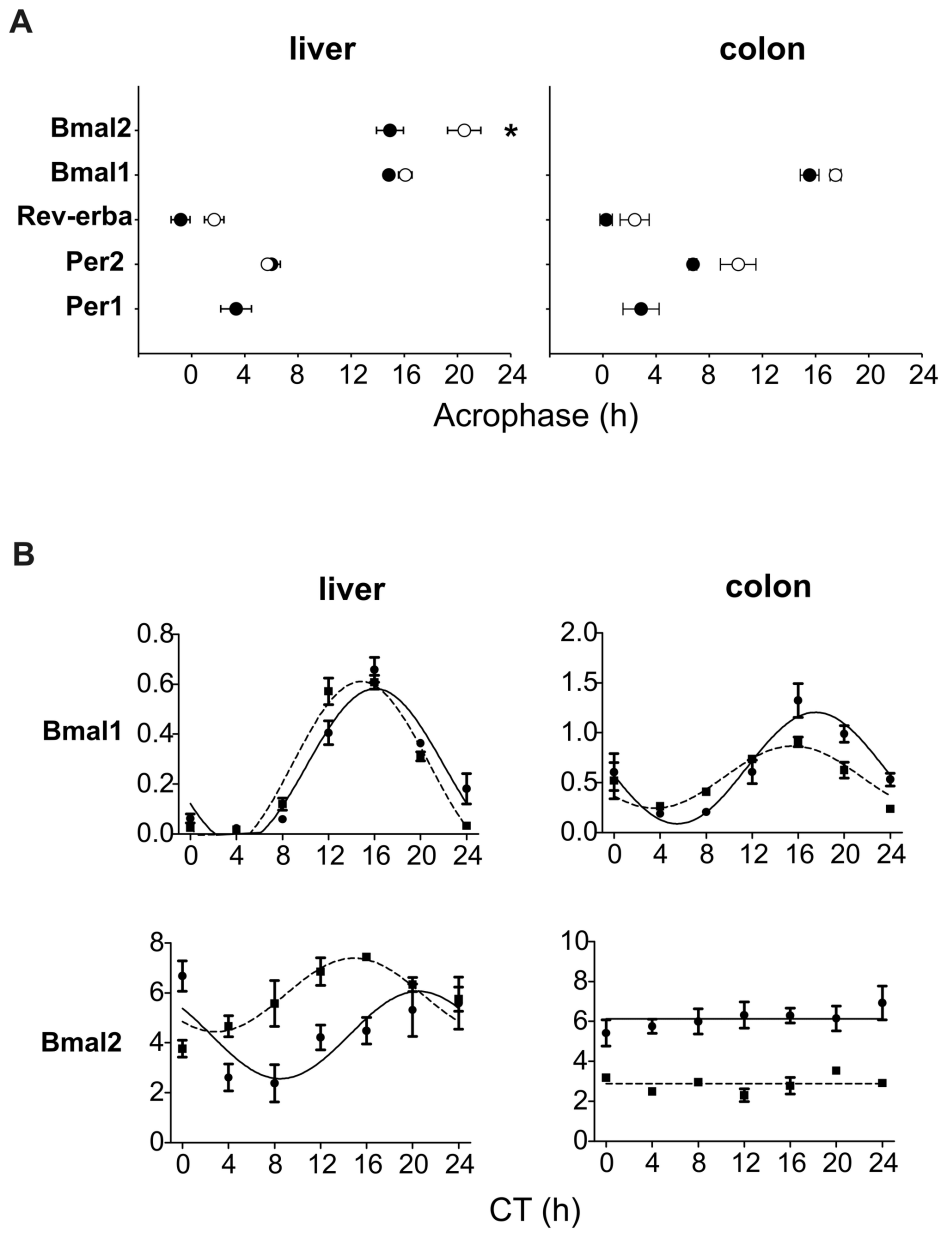
Comparison of the effect of restricted feeding on clock gene expression profiles in the liver and colon between the Wistar rats and SHR. The rats were maintained and sampled as described in Figure 2. For comparison, the mRNA levels of both rat strains were analyzed in the same qRT-PCR run. **A**) **Comparison of acrophases**. The acrophases were determined by cosine analysis of the *Per1*, *Per2*, *Rev-erbα*, *Bmal1* and *Bmal2* expression profiles determined in the liver (left) and colon (right) of Wistar rats (open circles) and SHR (full circles) maintained under LD12: 12 and subjected to a restricted feeding regime for 10 days. Each point represents the mean acrophase of the expression profile ± S.E.M. Data for *Per1* in the liver and colon of Wistar rats and for *Bmal2* in the colon of both rat strains are missing because these profiles did not exhibit a circadian rhythm. * marks statistically significant differences between the acrophases (P < 0.05). **B**) **Comparison of the *Bmal1* and *Bmal2* expression profiles**. The daily profiles of *Bmal1* and *Bmal2* expression in the liver (left) and colon (right) were determined in Wistar rats (open circles, full line) and SHR (full circles, dashed line) maintained under restricted feeding. Data are expressed as the relative expression; each point represents the mean ± S.E.M. of 3 (SHR) or 5 (Wistar rat) animals. For other details, see Figure 3.

To compare the characteristics (acrophases, mesors and amplitudes) of the clock gene expression profiles in the liver and colon between SHR and Wistar rats under RF, the clock gene expression profiles were assessed simultaneously in the same run of qRT-PCR for both strains. The results of the cosinor analysis are shown in Tables S4 and S5 and Figure 5.

The results revealed that the RF regime phase-advanced the peripheral clocks of the SHR more than those of the Wistar rats; the acrophases of the clock gene expression profiles were more advanced in the SHR in both the liver and the colon (Figure 5A) (apart from those which were arrhythmic, i.e., *Per1* in the liver and colon of Wistar rats and *Bmal2* in the colon of both strains, see Figures 3 and 4). The advance was statistically significant for *Bmal2* (t_54_ = -3.114, P = 0.05) in the liver but suggested also for other rhythmic genes (Figure 5a). In the liver, the expression profiles of *Per2* and *Bmal2* were up-regulated in the SHR compared with the Wistar rats (*Per2* mesor: t_54_ = 4.225, P < 0.001; *Bmal2* mesor: t_54_ = 3.733, P < 0.001), whereas in the colons of the SHR the profiles were down-regulated compared with those in the Wistar rats for *Per2* (mesor: t_52_ = -6.407, P < 0.001), *Rev-erbα* (mesor: t_53_ = -4,490, P < 0.001), and *Bmal2* (mesor: t_53_ = -10.631, P < 0.001). The clock gene expression profiles in the liver did not differ in amplitude between the strains; however, in the colon of the SHR, the circadian rhythm of *Bmal1* expression was suppressed (amplitude: t_54_ = -2.673, P = 0.010).

The data demonstrate that, under RF, the peripheral clocks in the liver and colon are more advanced in SHR than in Wistar rats. In SHR, the circadian expression of *Per2* and *Bmal2* is up-regulated in the liver and down-regulated in the colon compared with Wistar rats. Among all the studied clock genes, *Bmal2* exhibited the most significant differences in its daily expression profiles under RF between both strains and tissues. Moreover, the mutual phasing between *Bmal1* and *Bmal2* differed between the two rat strains (Figure 5B). In the liver, whereas in the Wistar rats the *Bmal2* profile was significantly delayed compared with *Bmal1*, the profiles of these two paralogs were in approximately the same phase in the SHR. In the colon, the *Bmal2* expression was arrhythmic in both strains and significantly down-regulated in the SHR compared with the Wistar rats.

Apart from Bmal2 mRNA, daily profiles of BMAL2 proteins levels were compared in the livers of Wistar rats and SHR subjected to RF (Figure S3). The data revealed that both strains differed in timing of the maximal BMAL2 levels. Whereas in Wistar rats the peak appeared during the end of subjective night, in SHR the peak was shifted to the subjective day.

### Comparison of the responsiveness of the clock - related gene expression profiles in the liver and colon to RF between in SHR and Wistar rats

Because the data described above suggest a higher sensitivity of the peripheral circadian clocks to RF in SHR compared with Wistar rats, the expression profiles of genes that are either under direct circadian control or whose protein products interact with the core clockwork (*Wee1*, *Dbp*, *E4bp4, Nampt*, *Ppara*, *Pparg*, *Pgc1α*, *Prkab2*, *Hdac3*, *Hif1a* and *Ppp1r3c*) were examined in the liver and colon of both rat strains under RF conditions (Tables S4 and S5). The daily expression profiles of all genes (with the exception of *Prkab2*) in both rat strains under *ad libitum* feeding conditions were described in our previous work [31].

**Figure 6:**
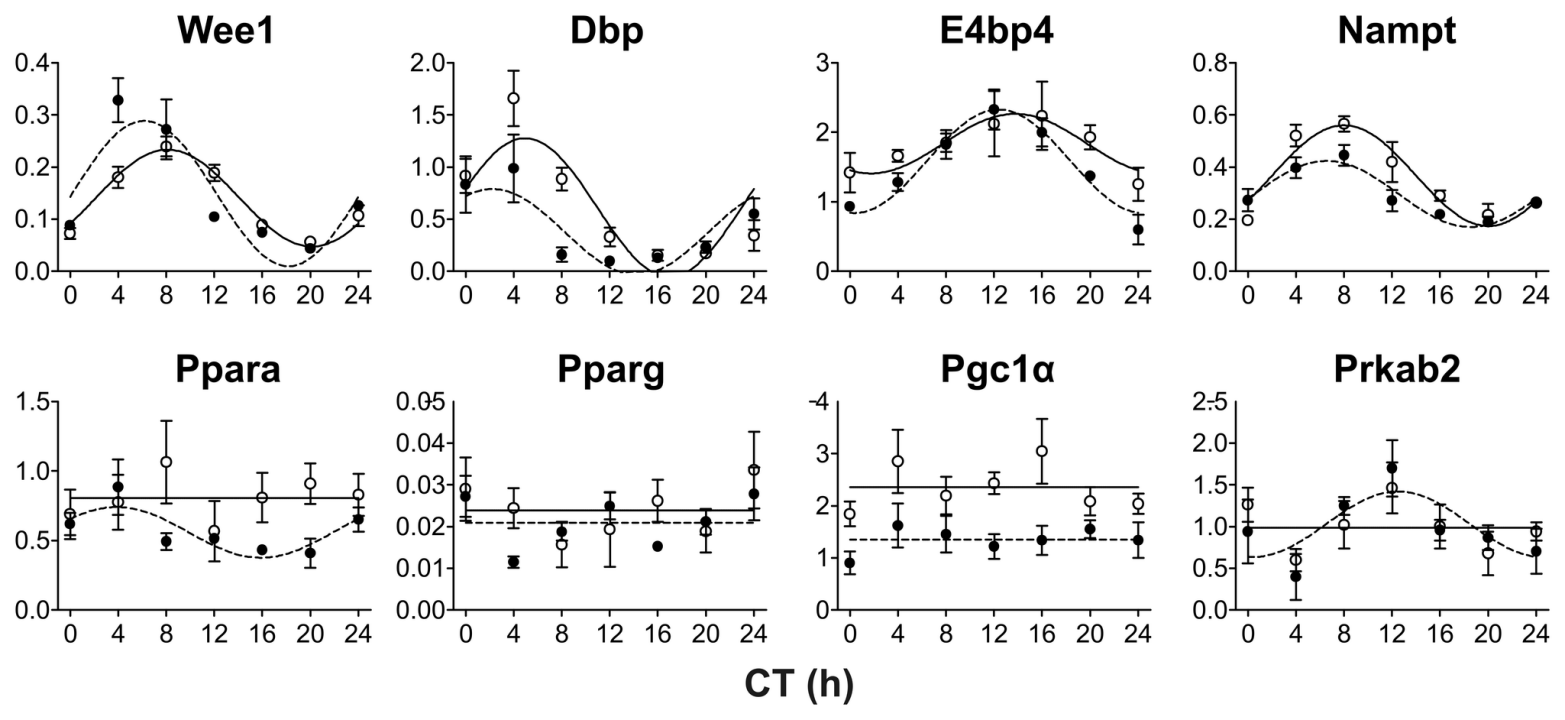
Comparison of the effect of restricted feeding on clock-controlled and clock-related gene expression profiles in the liver between the Wistar rats and the SHR. The rats were maintained and sampled as described in Figure 2. For comparison, the mRNA levels of both rat strains were analyzed in the same qRT-PCR run. The daily profiles of *Wee1*, *Dbp*, *E4bp4, Nampt*, *Ppara*, *Pparg*, *Pgc1α* and *Prkab2* expression were determined in the liver of Wistar rats (open circles, full line) and SHR (full circles, dashed line) maintained under restricted feeding. Data are expressed as the relative expression; each point represents the mean ± S.E.M. of 3 (SHR) or 5 (Wistar rat) animals. For other details, see Figure 3.

In the liver of the SHR (Figure 6), the circadian expression of the clock-controlled gene *Wee1* was significantly phase-advanced (acrophase: t_54_ = -2.745, P = 0.008) and its rhythmicity was enhanced (amplitude: t_53_ = 2.408, P = 0.020) compared with Wistar rats. These results were in agreement with our observations of advanced and upregulated clock gene expression profiles under RF in the SHR compared with the Wistar rats. However, under RF, the daily expression profiles of other studied genes in the SHR were either suppressed, namely *Dbp* (mesor: t_54_ = -2.224, P = 0.030), *Nampt* (mesor: t_54_ = -3.057, P = 0.003), *Ppara* (mesor: t_54_ = -2.358, P = 0.022) and *Pgc1α* (mesor: t_54_ = -4.727, P < 0.001), or did not differ (*E4bp4*, *Pparg*) when compared with those in the Wistar rats. The *Prkab2* expression exhibited significant rhythmicity in the SHR (P < 0.05) but was not rhythmic in the Wistar rats. The daily expression profiles of *Hdac3*, *Hif1a* and *Ppp1r3c* did not differ between the rat strains (data not shown).

In accordance with the above described suppression of the colonic clock in the SHR under a RF regime, the daily profiles of the majority of the clock- and metabolism-related genes studied herein were also suppressed in the SHR compared with the Wistar rats (Figure S2), namely *Wee1* (mesor: t_53_ = -3.014, P = 0.004), *Dbp* (mesor: t_53_ = -3.444, P = 0.001), *E4bp4* (mesor: t_54_ = -4.610, P < 0.001)*, Nampt* (mesor: t_53_ = -8.147, P < 0.001), *Pparg* (mesor: t_54_ = -4.366, P < 0.001), *Pgc1α* (mesor: t_54_ = -4.307, P < 0.001) and *Prkab2* (mesor: t_54_ = -5.893, P < 0.001). For *Ppara*, the suppression was only suggested. The expression profiles of *Hdac3*, *Hif1a* and *Ppp1r3c* were suppressed in the SHR compared with the Wistar rats: *Hdac3* (mesor: t_54_ = -2.932, P = 0.005), *Hif1a* (mesor: t_54_ = -4.440, P < 0.001), *Ppp1r3c* (mesor: t_54_ = -6.703, P < 0.001). Similar to the liver, the expression profile of *Wee1* in the colon of the SHR was significantly phase-advanced under RF compared with the Wistar rats (t_53_ = -3.664, P < 0.001). In contrast to the liver, *Pparg* was expressed rhythmically in the colon of both rat strains, and the profile in the SHR was also significantly phase advanced compared with the Wistar rats (t_54_ = -2.384, P = 0.021).

## Discussion

Our results demonstrate that SHR is behaviorally more sensitive to situations in which food availability is restricted to an improper time of day, developing earlier and stronger FAA than control Wistar rats. Whereas the restriction of food availability phase-shifted the nocturnal locomotor activity of the SHR, it only temporally redistributed this activity in the Wistar rats. The RF-induced behavioral phase shift in the SHR was not mediated by the SCN. Whereas RF exerted similar effects on the colon in both rat strains, it affected the hepatic clock of the SHR differently when compared with the Wistar rats. In the liver of the SHR, the expression profiles of *Bmal1* and *Bmal2* were synchronously advanced in response to RF, whereas in the Wistar rats only *Bmal1* expression was advanced. RF exerted a gene-, tissue- and strain-specific impact on the temporal regulation of the expression of genes that are either driven by the clock or whose protein products enable communication between the clock and metabolism. Altogether, these data suggest that the sensitivity of the circadian system to changes in the timing of food availability may greatly differ between the two closely related rat strains. Moreover, the results suggest a possible role of the *Bmal2* gene in the higher sensitivity of the hepatic clock of SHR to RF.

SHR/Ola exhibit a lean phenotype with body weight typically lower compared with Wistar rats. Under *ad libitum* feeding, SHR spent more time eating during the nighttime (65% of total hours) than during the daytime (35% of total hours). The percentage of time spent eating during the day seems to be higher than what we found previously in Wistar rats (cca 25%), which was in agreement with the findings of others [39]. This was also in agreement with previous observations of diminished diurnal variation in the feeding behavior of SHR compared with WKY rats as a result of increased food intake during the light period and mild decreases during dark periods [35]. It is possible that our result relates to our previous observation of a positive phase-angle of entrainment of the SCN-driven rhythms in SHR maintained on a LD cycle [31]. The rats became active prior to the onset of darkness and thus likely consumed more food during the daytime. During exposure to RF, the rats were allowed to consume food for only 6 h per day, which imposed a significant daily rhythm in the food intake of the animals. Moreover, the food was made available at improper times of day to increase the urgency of this signal. Nevertheless, the 6-h feeding regime likely represented sufficiently long interval to maintain normocaloric intake because rats of both strains gained weight during the 10 days of RF. Interestingly, under RF SHR consumed significantly more food and gained slightly more weight relative to their body weight than Wistar rats. Therefore, the observed increase in sensitivity to RF in SHR was not due to a change in caloric intake on RF. Our data demonstrate that the exposure of rats to RF induces FAA in both rat strains. However, the FAA occurred much earlier and was more pronounced in the SHR than in the Wistar rats, suggesting that for SHR, RF may present a stronger signal to entrain the putative food entrainable oscillator that drives FAA. In accordance with the finding of stronger FAA in SHR, the locomotor activity under RF was completely phase-advanced according to the time of food availability. In contrast, the locomotor activity of the Wistar rats was redistributed due to RF into only two bouts, one related to food presence and the second remaining synchronized with the external LD cycle. A similar effect of RF on the locomotor activity of Wistar rats has been observed previously elsewhere (e.g., see [40]). The results suggest that whereas in Wistar rats the behavioral activity is entrained by RF as well as by the external LD conditions, in SHR, RF dominates the entraining cue. Thus, our data clearly demonstrate that RF is a much stronger entraining cue for SHR than for Wistar rats; however, the underlying mechanism is not clear. Nevertheless, based on the published data, it is plausible to speculate that brain active mediators such as ghrelin and/or orexin may play a role in FAA. Their involvement in the mediation of FAA has previously been suggested because their deficiency resulted in decreased FAA in mice (reviewed by [41]). Interestingly, ghrelin plasma levels [42] as well as orexinergic activity in the brain [43] were found to be elevated in SHR compared with controls. It remains to be tested whether these anomalies may contribute to the higher FAA response to RF in this rat strain.

To test the hypothesis that the enhanced food anticipatory activity might result from a higher sensitivity of the circadian system to changes in feeding conditions, we determined the daily profiles of clock gene expression in the SCN and peripheral clocks of SHR and Wistar rats. Our previous data demonstrated that under *ad libitum* feeding conditions, the circadian system of SHR exhibits distinct differences when compared with that of Wistar rats [31]. Specifically, the SHR exhibited a positive phase angle of entrainment of the locomotor activity rhythms that was likely due to a phase-advanced SCN clock. The current data demonstrate that the phasing of the clock gene expression profiles in the SCN of SHR is not affected by RF, a result which is in good agreement with our previous findings in Wistar rats [29] as well as with findings in all other species studied so far. Therefore, the higher sensitivity of SHR to RF, as reflected by the stronger FAA and phase advances of the locomotor activity in SHR, was not mediated by the SCN. Nevertheless, the question remains whether the positive phase angle of entrainment under *ad libitum* conditions in SHR may contribute to the effect of RF on the phasing of their locomotor activity.

RF has been widely recognized as a strong entraining signal to some peripheral clocks, including those in the liver [26,27,28] and colon [29,30]. Our previous data revealed that the phasing and amplitude of the circadian clock oscillation in the liver did not differ between SHR and Wistar rats under *ad libitum* conditions, whereas the clock in the colon was advanced and dampened [31]. In the present study, RF significantly phase advanced the daily profiles of clock gene expression in both peripheral tissues according to the time of food presentation in both rat strains. The colonic clock responded to RF in a very similar manner in both rat strains. However, obvious strain-dependent differences in the response to RF were detected in the liver. Whereas RF suppressed the oscillation of clock gene expression in the liver of the Wistar rats, no such suppression was detected in the SHR. Furthermore, in the SHR the amplitude of the *Per2* expression rhythm increased significantly in response to RF. These results demonstrate that the oscillation of the hepatic clock is facilitated in SHR exposed to RF. The most striking difference between the two rat strains was found in the effect of RF on the temporal control of the *Bmal2* mRNA profiles. In the Wistar rats, the *Bmal2* expression did not exhibit circadian variation under *ad libitum* conditions and became expressed rhythmically with a very low amplitude under RF. However, the low-amplitude *Bmal2* oscillation was not in phase with *Bmal1* under RF and instead remained in approximately the same phase as *Bmal1* under the *ad libitum* feeding conditions. Therefore, it is uncertain whether RF indeed phase-shifted *Bmal2* expression in the liver of the Wistar rats. In contrast, in the SHR, *Bmal2* was expressed with a low amplitude under *ad libitum* conditions, and the amplitude of the rhythm increased and was significantly phase advanced under RF. Importantly, in the SHR, both *Bmal* paralogs were in the same phase under RF. These data demonstrate a higher sensitivity of the *Bmal2* gene to RF in the liver of SHR. The marked response of *Bmal2* correlated with the enhanced amplitude of the *Per2* rhythm in the SHR under RF. These data are in accordance with a functional partnership between BMAL2 and PER2 [44]. Indeed, daily temporal regulation of BMAL2 protein levels seemed also to differ between SHR and Wistar rats under RF; in SHR under RF, the highest BMAL2 levels were detected during subjective day when *Per2* transcription was increased compared with Wistar rats. Hence, in SHR maintained under RF, BMAL2-CLOCK-mediated transactivation may support the conventional BMAL1-CLOCK mediated pathways, leading to an increased clock oscillation amplitude, as demonstrated in our study. Consequently, the higher amplitude of the *Per2* expression rhythm might be related to higher levels of the corresponding protein that facilitates PER2-mediated inhibition of the BMAL2-CLOCK. This mechanism is likely not present in Wistar rats, in which the *Bmal2* gene does not seem to be sensitive to RF, and the clock amplitude is thus suppressed under RF conditions. *Bmal2* is hypothesized to have been generated by gene duplication at the beginning of vertebrate evolution [45]. In early studies, the role of *Bmal2* in the circadian clock mechanism was not recognized [46] because it was downregulated in *Bmal1*-deficient mice together with its paralog. However, recently accumulated evidence [7,44] and our current data suggest its plausible physiological role in mediating the changes in feeding regimes by the hepatic clock. Our hypothesis that *Bmal2* plays a role in mediating the interaction between the clock and metabolism is further supported by a recent finding that the constitutive expression of *Bmal2* rescues the rhythmicity of the insulin action, locomotor activity and oxygen consumption in *Bmal1*-knockout mice [7,47]. The *Bmal2* gene is considered to be associated with type 1 diabetes in non-obese mice [48]. Moreover, it has been suggested that alternative splicing of *Bmal2* may provide tissues with a pathway regulating CLOCK-BMAL2 heterodimer function to respond to varied metabolic demands [49]. In addition, BMAL2 was found to selectively bind to the E-box on the promoter of *Pai-1* and regulate its expression [50]. *Pai-1* is elevated and associated with metabolic syndrome [51]. Hence, a role of BMAL2 in mediating the interaction between the clock and metabolic state is likely, and our current data suggest that the interaction is likely bi-directional. The strain-selective sensitivity of *Bmal2* to RF in SHR might be related to i) polymorphisms of the gene (or its promoter region), and/or ii) to a higher metabolic challenge impinging on the clock in the rat strain. The former possibility is unlikely because the *Bmal2* coding sequence in SHR and Wistar rats does not differ [31], although there might be undocumented polymorphisms in the regulatory regions. Several polymorphisms associated with metabolic syndrome were identified in the SHR *Bmal1* promoter [34]. Two of the SNPs affected the transcriptional binding sites for the GATA (NF-E1) and Pax6 (paired box gene 6) nuclear factors and significantly lowered the promoter activity. Therefore, it is possible that this deficiency is compensated by the higher *Bmal2* sensitivity in SHR. The latter possibility of a higher metabolic pressure on the clock in SHR is supported by the previously demonstrated relationship between hypertension and hepatic physiology, as manifested by the metabolic aberrances of SHRs [32] and the differences in the expression of metabolism-relevant proteins in SHR liver [52], as well as by the different sensitivity of the metabolic markers to RF, as discussed below.

The higher amplitude and the advance of the hepatic clock rhythmicity under RF in the SHR compared with the Wistar rats corresponded to an enhanced and phase-advanced rhythmicity of *Wee1* expression. WEE1 is a kinase which inactivates the complex Cdc2/cyclin B, thus controlling the G_2_/M transition of the cell cycle in a circadian manner [53]. We used this gene as a marker of conventional E-box driven rhythmicity to confirm the effect of RF on the circadian clock in SHR. Importantly, under *ad libitum* feeding conditions, the *Wee1* expression profiles in SHR liver did not differ from those in Wistar rats [31]. For the other E-box driven genes studied herein, namely those involved in interactions between the clock and metabolism, i.e., *Dbp* (transcription factor regulating many downstream genes, e.g., detoxification enzymes [54,55]), *E4bp4* (transcriptional repressor of many downstream genes [56,57], also involved in regulation of *Per2* transcription [9]) and *Nampt* (rate limiting enzyme in biosynthesis of NAD^+^ [58,59]), the advance was subtle and the amplitude was only slightly decreased. Similarly to that of Wee1, the expression profile of none of these genes did differ between the two rat strains under *ad libitum* feeding [31]. Under RF conditions, the expression of *Prkab2* (the regulatory beta 2 subunit of AMP activated protein kinase [60]) exhibited a circadian oscillation in the SHR but was not rhythmic in the Wistar rats. The results also demonstrated that under RF, expression profiles of some studied genes in the liver of SHR were suppressed or did not differ compared with those in Wistar rats. This might be because these genes are not driven by the clock but rather by changes in the metabolic state. The RF likely affected the metabolic state of the SHR differently than the Wistar rats because the constitutive expression of *Pgc1α*, which coordinates the transcriptional programs important for energy metabolism and homeostasis [8], was significantly suppressed in the SHR compared with the Wistar rats. *Pgc1α* encodes a co-activator that enhances the activity of many nuclear receptors, including PPARα (a transcription factor involved in the regulation of adipogenesis, lipid and glucose homeostasis, linked to circadian clock [61]). It is noteworthy that the *Ppara* expression was also slightly suppressed in the SHRs but became expressed rhythmically in response to RF, though with a low amplitude. The observed suppression was not due to a lower strain-specific basal expression of these genes under *ad libitum* conditions [31]. The significant suppression of *Pgc1α* and *Ppara* expression may likely result in decreased levels of the PPARα/PGC1α transcriptional complex with nuclear receptors, which may consequently affect the activity of downstream genes regulating fatty acid uptake and oxidation in the liver of SHR maintained under RF. The other metabolism-relevant gene expression profiles in the liver did not differ between the two strains, namely that of *Pparg* (critical regulator of adipogenesis and osteogenesis [62,63]), *Hdac3* (histone deacetylase integrating metabolic and circadian regulation [64,65]), *Hif1a* (the alpha subunit of the hypoxia inducible factor regulating homeostatic response to hypoxia [66]) and *Ppp1r3c* (a subunit of protein phosphatase 1 and major metabolic regulator linked to the circadian clock [67,68]). Thus, the data suggest that RF impacts the metabolic state in the liver of SHR differently than in Wistar rats, with the most obvious difference in the suppression of *Pgc1α* and *Ppara* expression and the imposition of a low-amplitude rhythmicity in *Ppara* and *Prkab2* expression. These changes in gene expression might have a broad impact on the transcription of variety of genes, including those whose products feed back on the circadian clock mechanism (see above). We hypothesize that as a consequence, apart from other effects, the transcriptional activity of the *Bmal2* gene may also have changed. Based on the limited data available, the likely pathways for this effect are highly speculative. To test the hypothesis that the abnormal metabolic state of SHR under RF may account for the higher sensitivity of their hepatic circadian clock to changes in the feeding regime, future studies should examine the suggested pathways at the level of protein expression and activities. It is also reasonable to consider the involvement of glucocorticoids, which are known to play a role in resetting the phase of the peripheral clock in the liver [69] and whose peak levels are shifted due to RF in rats [14,70]. The general hypersensitivity of the hypothalamic-pituitary-adrenal system of SHR [71,72] might theoretically account for the facilitation of the hepatic clock response to RF. However, similar to the previously suggested pathways, the role of glucocorticoids in this effect has not been tested.

In conclusion, our results demonstrate a previously unknown correlation between the extent of FAA and the phase-shifting of the peripheral clock in the liver. Additionally, our evidence that the putative food-entrainable oscillator driving the FAA is more sensitive in SHR may facilitate future studies aimed at localizing this clock in the mammalian body. Our results also suggest a possible role of the *Bmal2* gene in phase-shifting the hepatic clock in response to RF in SHR. Finally, these results demonstrate the complexity of the circadian phenotype of SHR pointing at the mutual relationship between the circadian system and metabolism as well as at the strain-dependent changes involved in the response to external challenges, such as a temporal change in food availability.

## Supporting Information

Figure S1 **Body weight and food consumption during RF.** Animals were subjected to RF as described in Figure 1A. **A**) Body weight was recorded before the start of RF (yellow), after 5 days of RF (orange), and after 10 days of RF (red). **B**) Food consumption was detected every day of RF. Absolute amount of food consumed during the RF was measured for Wistar rats (pink open circles and full line) and SHR (green full circles and dashed line). Food consumption relative to body weight (g/kg of initial body weight) during RF was calculated for Wistar rats (black open circles and full line) and SHR (black full circles and dashed line). **C**) Absolute weight gain was measured in Wistar rats (open column) and SHR (black column) as a difference between the body weight after and before RF; **D**) Relative weight gain was detected in Wistar rats (open column) and SHR (black column) by normalizing the values from C to the initial body weight. Data represent a mean ± S.D. of 5 animals. The asterisks show the results of two-way ANOVA with Bonferroni multiple comparisons test (* P < 0.05, ** P < 0.01 and *** P < 0.001).
(TIF)

Figure S2 **Comparison of the effect of restricted feeding on clock-controlled and clock-related gene expression profiles in the colon between the Wistar rats and SHR.** The rats were maintained and sampled as described in Figure 2. For comparison, the mRNA levels of both rat strains were analyzed in the same qRT-PCR run. The daily profiles of *Wee1*, *Dbp*, *E4bp4, Nampt*, *Ppara*, *Pparg*, *Pgc1α* and *Prkab2* expression were determined in the liver of Wistar rats (open circles, full line) and SHR (full circles, dashed line) maintained under restricted feeding. Data are expressed as the relative expression; each point represents the mean ± S.E.M. of 3 (SHR) or 5 (Wistar rat) animals. Time is expressed as circadian time (CT), with CT0 corresponding to lights-on in the previous LD cycle. Data were fit with cosine curves to calculate the acrophases, amplitudes and mesors (for details, see Methods).
(TIF)

Figure S3 **BMAL2 protein level in the liver of SHR and Wistar rats exposed to RF.** Animals were subjected to RF as described in Figure 1A and killed in 6-h intervals during the 24 h. Each data point represents one animal. **A**) **Western blot of liver** **BMAL2** and β-actin loading control. The circadian time is depicted above. Wistar rat (left) and SHR (right) samples were run on a single gel. **B**) **Relative BMAL2 protein level**. The western blot data of Wistar rat (open circles and full line) and SHR (full circles and dashed line) BMAL2 levels from **A**) were normalized to β-actin. Note the rhythmic expression and the different phase of BMAL2 liver protein in both rat strains.
(TIF)

Table S1 **Cosinor analysis of SCN expression profiles.**
(DOCX)

Table S2 **Cosinor analysis of expression profiles a) in Wistar liver; b) in SHR liver.**
(DOCX)

Table S3 **Cosinor analysis of expression profiles a) in Wistar colon; b) in SHR colon.**
(DOCX)

Table S4 **Cosinor analysis of liver expression profiles on RF.**
(DOCX)

Table S5 **Cosinor analysis of colon expression profiles on RF.**
(DOCX)
