# Supplementary figures and images for: Increased Sensitivity of the Circadian System to Temporal Changes in the Feeding Regime of Spontaneously Hypertensive Rats - A Potential Role for Bmal2 in the Liver
Source: PLoS One. 2013 Sep 25;8(9):e75690. doi: 10.1371/journal.pone.0075690 (PMC3783415; doi:10.1371/journal.pone.0075690)

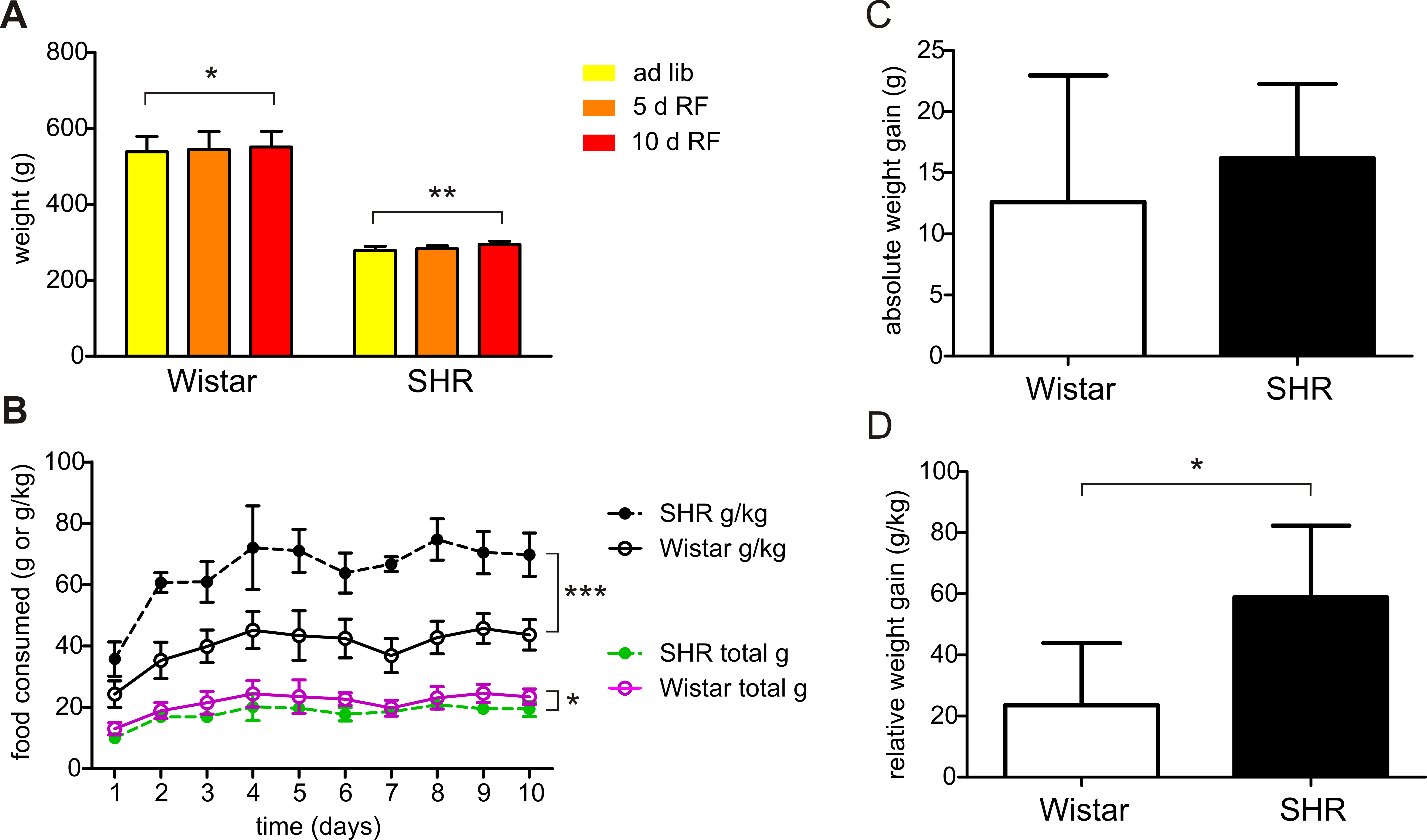

Supplement: Figure S1 — Body weight and food consumption during RF. Animals were subjected to RF as described in Figure 1A. A) Body weight was recorded before the start of RF (yellow), after 5 days of RF (orange), and after 10 days of RF (red). B) Food consumption was detected every day of RF. Absolute amount of food consumed during the RF was measured for Wistar rats (pink open circles and full line) and SHR (green full circles and dashed line). Food consumption relative to body weight (g/kg of initial body weight) during RF was calculated for Wistar rats (black open circles and full line) and SHR (black full circles and dashed line). C) Absolute weight gain was measured in Wistar rats (open column) and SHR (black column) as a difference between the body weight after and before RF; D) Relative weight gain was detected in Wistar rats (open column) and SHR (black column) by normalizing the values from C to the initial body weight. Data represent a mean ± S.D. of 5 animals. The asterisks show the results of two-way ANOVA with Bonferroni multiple comparisons test (* P < 0.05, ** P < 0.01 and *** P < 0.001). (TIF) [file pone.0075690.s001.tif]

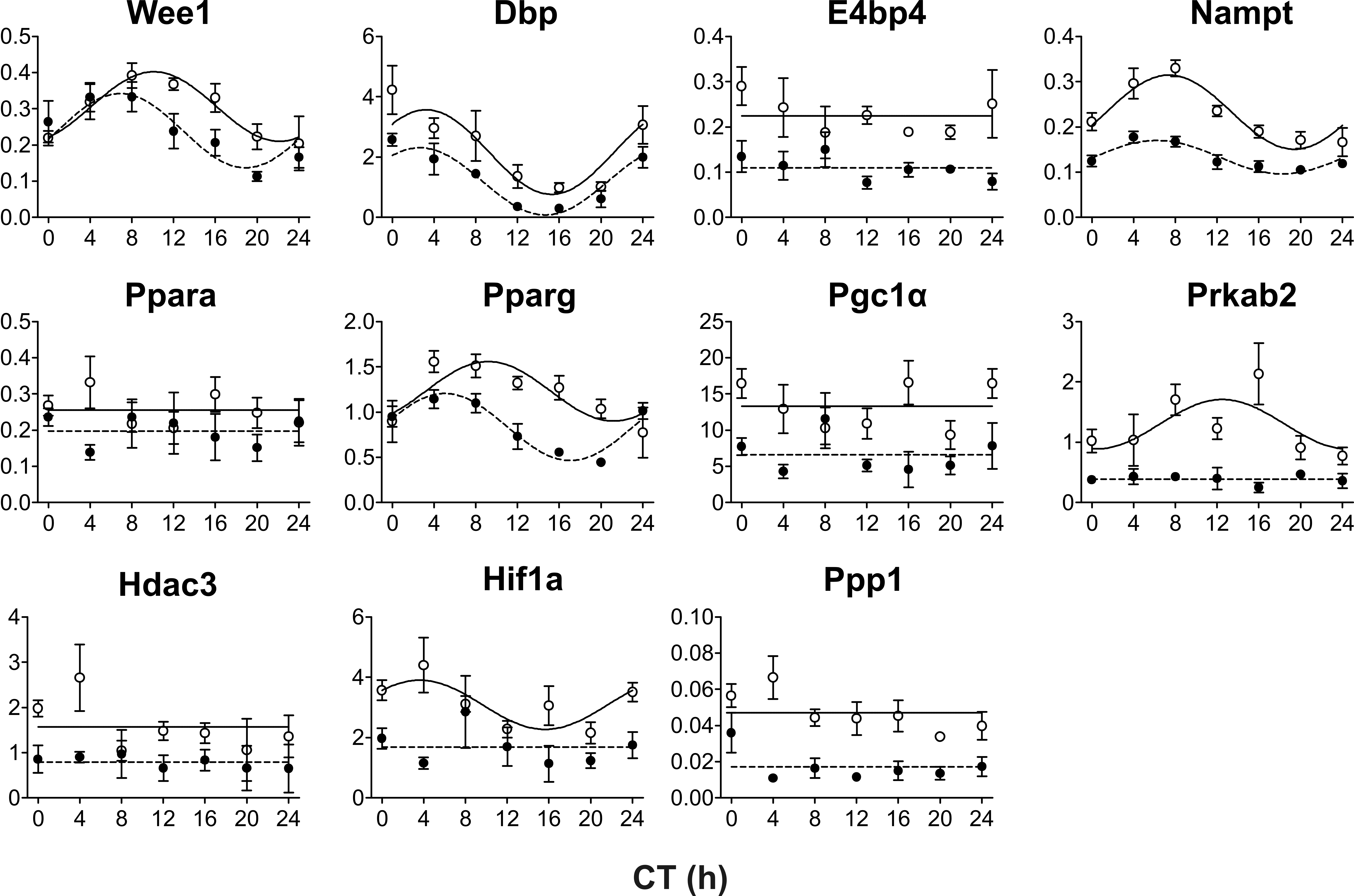

Supplement: Figure S2 — Comparison of the effect of restricted feeding on clock-controlled and clock-related gene expression profiles in the colon between the Wistar rats and SHR. The rats were maintained and sampled as described in Figure 2. For comparison, the mRNA levels of both rat strains were analyzed in the same qRT-PCR run. The daily profiles of Wee1, Dbp, E4bp4, Nampt, Ppara, Pparg, Pgc1α and Prkab2 expression were determined in the liver of Wistar rats (open circles, full line) and SHR (full circles, dashed line) maintained under restricted feeding. Data are expressed as the relative expression; each point represents the mean ± S.E.M. of 3 (SHR) or 5 (Wistar rat) animals. Time is expressed as circadian time (CT), with CT0 corresponding to lights-on in the previous LD cycle. Data were fit with cosine curves to calculate the acrophases, amplitudes and mesors (for details, see Methods). (TIF) [file pone.0075690.s002.tif]

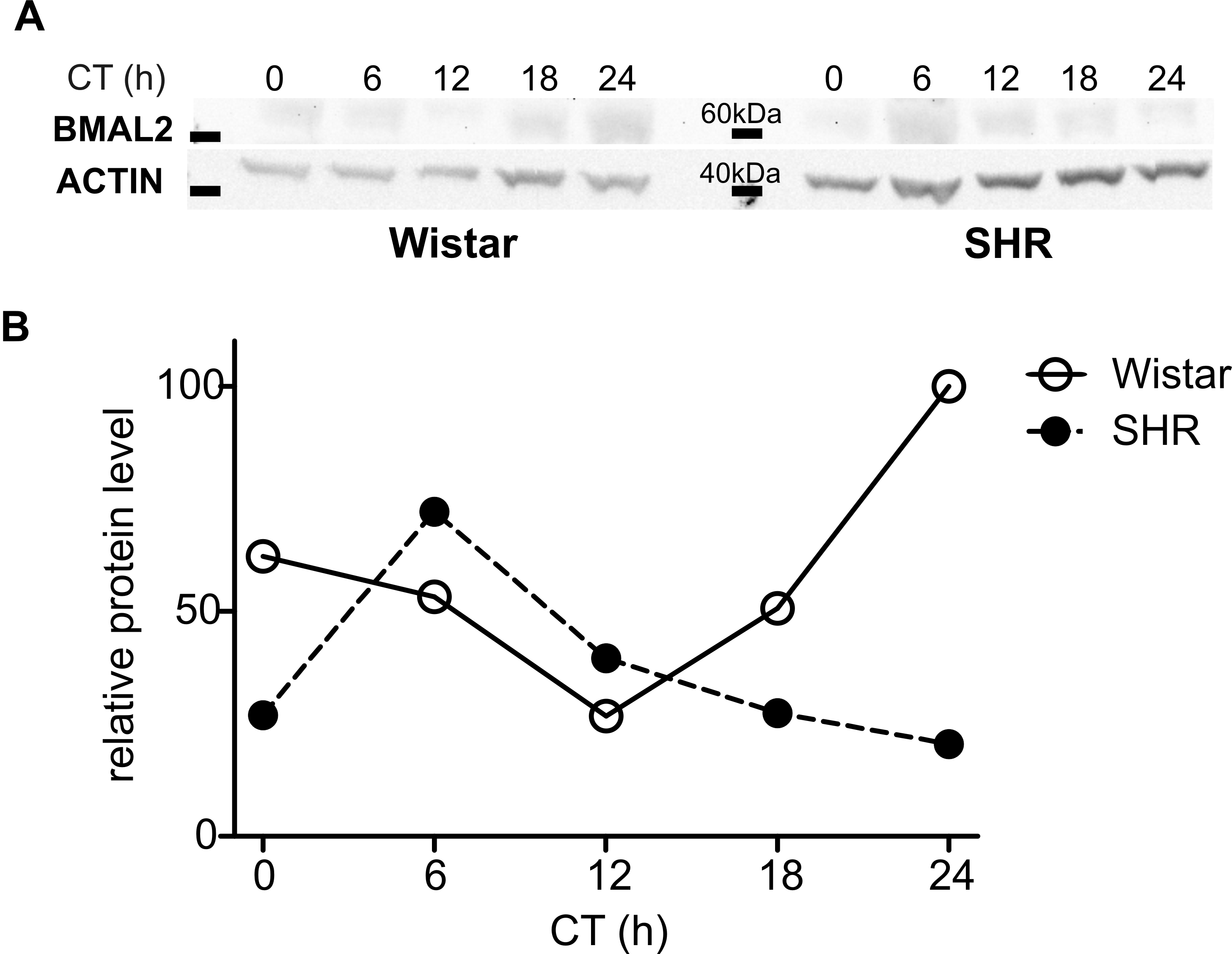

Supplement: Figure S3 — BMAL2 protein level in the liver of SHR and Wistar rats exposed to RF. Animals were subjected to RF as described in Figure 1A and killed in 6-h intervals during the 24 h. Each data point represents one animal. A) Western blot of liver BMAL2 and β-actin loading control. The circadian time is depicted above. Wistar rat (left) and SHR (right) samples were run on a single gel. B) Relative BMAL2 protein level. The western blot data of Wistar rat (open circles and full line) and SHR (full circles and dashed line) BMAL2 levels from A) were normalized to β-actin. Note the rhythmic expression and the different phase of BMAL2 liver protein in both rat strains. (TIF) [file pone.0075690.s003.tif]
