# Supplementary material for: Increased Sensitivity of the Circadian System to Temporal Changes in the Feeding Regime of Spontaneously Hypertensive Rats - A Potential Role for Bmal2 in the Liver
Source: PLoS One. 2013 Sep 25;8(9):e75690. doi: 10.1371/journal.pone.0075690 (PMC3783415; doi:10.1371/journal.pone.0075690)
Supplement: Table S1 — Cosinor analysis of SCN expression profiles. (DOCX) [file pone.0075690.s004.docx]

Table S1. Cosinor analysis of SCN expression profiles

|  | **Gene** | **Per2** | | **Rev-erbα** | | **Bmal1** | |
| --- | --- | --- | --- | --- | --- | --- | --- |
|  | **Feeding** | **ad lib** | **RF** | **ad lib** | **RF** | **ad lib** | **RF** |
| **P value** | | < 0.0001 | < 0.0001 | < 0.0001 | < 0.0001 | < 0.0001 | < 0.0001 |
| **R^2^** | | 0.9155 | 0.9273 | 0.9108 | 0.9246 | 0.6388 | 0.8356 |
| **Mesor** | | 0.1552 | 0.1370 | 0.1701 | 0.1912 | 0.1040 | 0.0987 |
| **SE Mesor** | | 0.0057 | 0.0058 | 0.0052 | 0.0044 | 0.0052 | 0.0037 |
| **Amplitude** | | 0.1189 | 0.1218 | 0.0985 | 0.0876 | 0.0431 | 0.0538 |
| **SE Amplitude** | | 0.0084 | 0.0083 | 0.0071 | 0.0061 | 0.0075 | 0.0056 |
| **Acrophase** | | 8.05 | 8.27 | 2.23 | 2.72 | 15.78 | 17.90 |
| **SE Acrophase** | | 0.25 | 0.25 | 0.29 | 0.27 | 0.63 | 0.35 |

R^2^ (coefficient of determination)
