# Supplementary material for: Increased Sensitivity of the Circadian System to Temporal Changes in the Feeding Regime of Spontaneously Hypertensive Rats - A Potential Role for Bmal2 in the Liver
Source: PLoS One. 2013 Sep 25;8(9):e75690. doi: 10.1371/journal.pone.0075690 (PMC3783415; doi:10.1371/journal.pone.0075690)
Supplement: Table S2 — Cosinor analysis of expression profiles a) in Wistar liver; b) in SHR liver. (DOCX) [file pone.0075690.s005.docx]

Table S2a. Cosinor analysis of expression profiles in Wistar liver.

| **Gene** | **Per1** | **Per1** | **Per2** | **Per2** | **Rev-erbα** | **Rev-erbα** | **Bmal1** | **Bmal1** | **Bmal2** | **Bmal2** |
| --- | --- | --- | --- | --- | --- | --- | --- | --- | --- | --- |
| **Feeding** | ad lib | RF | ad lib | RF | ad lib | RF | ad lib | RF | ad lib | RF |
| **P value** | 0.0003 | 0.2087 | < 0.0001 | < 0.0001 | < 0.0001 | < 0.0001 | < 0.0001 | < 0.0001 | 0.2529 | 0.0466 |
| **R^2^** | 0.5928 | 0.0933 | 0.7949 | 0.5465 | 0.8160 | 0.6517 | 0.9196 | 0.7100 | 0.1417 | 0.1794 |
| **Mesor** | 0.1013 | 0.1165 | 0.4314 | 0.2375 | 0.8634 | 0.7106 | 0.4181 | 0.2396 | 7.6200 | 8.8390 |
| **SE Mesor** | 0.0110 | 0.0141 | 0.0193 | 0.0111 | 0.1013 | 0.0838 | 0.0214 | 0.0191 | 0.4586 | 0.5581 |
| **Amplitude** | 0.0772 |  | 0.2364 | 0.1029 | 1.2770 | 0.8762 | 0.4073 | 0.2486 |  | 1.9490 |
| **SE Amplitude** | 0.0152 |  | 0.0284 | 0.0166 | 0.1441 | 0.1138 | 0.0284 | 0.0282 |  | 0.7533 |
| **Acrophase** | 14.24 |  | 16.35 | 5.24 | 8.84 | 1.71 | 0.49 | 16.48 |  | 22.11 |
| **SE Acrophase** | 0.79 |  | 0.42 | 0.55 | 0.43 | 0.54 | 0.30 | 0.40 |  | 1.63 |

R^2^ (coefficient of determination)

Table S2a. Cosinor analysis of expression profiles in SHR liver.

| **Gene** | **Per1** | **Per1** | **Per2** | **Per2** | **Rev-erbα** | **Rev-erbα** | **Bmal1** | **Bmal1** | **Bmal2** | **Bmal2** |
| --- | --- | --- | --- | --- | --- | --- | --- | --- | --- | --- |
| **Feeding** | ad lib | RF | ad lib | RF | ad lib | RF | ad lib | RF | ad lib | RF |
| **P value** | < 0.0001 | 0.0007 | < 0.0001 | < 0.0001 | < 0.0001 | < 0.0001 | < 0.0001 | < 0.0001 | 0.0032 | 0.0029 |
| **R^2^** | 0.7591 | 0.5518 | 0.8851 | 0.7901 | 0.8484 | 0.8679 | 0.9358 | 0.9514 | 0.4712 | 0.4773 |
| **Mesor** | 0.1288 | 0.1366 | 0.2801 | 0.3602 | 0.5865 | 0.6548 | 0.3393 | 0.3255 | 2.2620 | 3.213 |
| **SE Mesor** | 0.0112 | 0.0143 | 0.0125 | 0.0223 | 0.0604 | 0.0565 | 0.0174 | 0.0144 | 0.0882 | 0.1244 |
| **Amplitude** | 0.1115 | 0.0972 | 0.2084 | 0.2749 | 0.8829 | 0.8143 | 0.3761 | 0.3754 | 0.4825 | 0.6956 |
| **SE Amplitude** | 0.0148 | 0.0208 | 0.0178 | 0.0334 | 0.0884 | 0.0749 | 0.0233 | 0.0202 | 0.1213 | 0.1728 |
| **Acrophase** | 11.55 | 3.93 | 15.21 | 6.10 | 7.84 | 23.51 | 22.91 | 14.73 | 21.85 | 14.45 |
| **SE Acrophase** | 0.57 | 0.77 | 0.32 | 0.41 | 0.36 | 0.40 | 0.26 | 0.21 | 1.01 | 0.98 |

R^2^ (coefficient of determination)
