# Supplementary material for: Increased Sensitivity of the Circadian System to Temporal Changes in the Feeding Regime of Spontaneously Hypertensive Rats - A Potential Role for Bmal2 in the Liver
Source: PLoS One. 2013 Sep 25;8(9):e75690. doi: 10.1371/journal.pone.0075690 (PMC3783415; doi:10.1371/journal.pone.0075690)
Supplement: Table S3 — Cosinor analysis of expression profiles a) in Wistar colon; b) in SHR colon. (DOCX) [file pone.0075690.s006.docx]

Table S3a. Cosinor analysis of expression profiles in Wistar colon.

| **Gene** | **Per1** | **Per1** | **Per2** | **Per2** | **Rev-erbα** | **Rev-erbα** | **Bmal1** | **Bmal1** | **Bmal2** | **Bmal2** |
| --- | --- | --- | --- | --- | --- | --- | --- | --- | --- | --- |
| **Feeding** | ad lib | RF | ad lib | RF | ad lib | RF | ad lib | RF | ad lib | RF |
| **P value** | 0.0024 | 0.0167 | 0.0019 | < 0.0001 | < 0.0001 | < 0.0001 | < 0.0001 | < 0.0001 | 0.0839 | 0.1977 |
| **R^2^** | 0.3149 | 0.2536 | 0.3233 | 0.4606 | 0.7951 | 0.5429 | 0.8993 | 0.8464 | 0.1435 | 0.0993 |
| **Mesor** | 0.3645 | 0.3693 | 0.8552 | 1.0290 | 0.7975 | 1.0040 | 1.1660 | 0.8391 | 31.9800 | 23.2500 |
| **SE Mesor** | 0.0242 | 0.0235 | 0.0497 | 0.0426 | 0.0511 | 0.0680 | 0.0332 | 0.0316 |  |  |
| **Amplitude** | 0.1233 | 0.1059 | 0.2905 | 0.2970 | 0.7700 | 0.5812 | 0.7718 | 0.6278 |  |  |
| **SE Amplitude** | 0.0322 | 0.0344 | 0.0743 | 0.0579 | 0.0695 | 0.0950 | 0.0460 | 0.0473 |  |  |
| **Acrophase** | 12.66 | 6.96 | 17.43 | 10.62 | 10.23 | 2.64 | 2.34 | 17.55 |  |  |
| **SE Acrophase** | 1.12 | 1.14 | 0.87 | 0.81 | 0.37 | 0.64 | 0.24 | 0.25 |  |  |

R^2^ (coefficient of determination)

Table S3b. Cosinor analysis of expression profiles in SHR colon.

| **Gene** | **Per1** | **Per1** | **Per2** | **Per2** | **Rev-erbα** | **Rev-erbα** | **Bmal1** | **Bmal1** | **Bmal2** | **Bmal2** |
| --- | --- | --- | --- | --- | --- | --- | --- | --- | --- | --- |
| **Feeding** | ad lib | RF | ad lib | RF | ad lib | RF | ad lib | RF | ad lib | RF |
| **P value** | 0.0002 | 0.0003 | < 0.0001 | < 0.0001 | < 0.0001 | < 0.0001 | < 0.0001 | < 0.0001 | 0.1292 | 0.5233 |
| **R^2^** | 0.6037 | 0.5898 | 0.8389 | 0.8643 | 0.8654 | 0.8027 | 0.8264 | 0.7220 | 0.2034 | 0.0694 |
| **Mesor** | 0.1226 | 0.0988 | 0.4868 | 0.4119 | 0.4005 | 0.3753 | 0.3298 | 0.3146 | 10.4400 | 8.2130 |
| **SE Mesor** | 0.0083 | 0.0085 | 0.0228 | 0.0182 | 0.0319 | 0.0311 | 0.0247 | 0.0234 |  |  |
| **Amplitude** | 0.0577 | 0.0616 | 0.3191 | 0.2907 | 0.4820 | 0.3516 | 0.3027 | 0.2291 |  |  |
| **SE Amplitude** | 0.0110 | 0.0122 | 0.0332 | 0.0272 | 0.0452 | 0.0411 | 0.0327 | 0.0337 |  |  |
| **Acrophase** | 11.66 | 3.57 | 15.86 | 6.87 | 8.95 | 0.00 | 0.18 | 15.68 |  |  |
| **SE Acrophase** | 0.83 | 0.73 | 0.38 | 0.32 | 0.36 | 0.51 | 0.47 | 0.54 |  |  |

R^2^ (coefficient of determination)
