# Supplementary material for: Increased Sensitivity of the Circadian System to Temporal Changes in the Feeding Regime of Spontaneously Hypertensive Rats - A Potential Role for Bmal2 in the Liver
Source: PLoS One. 2013 Sep 25;8(9):e75690. doi: 10.1371/journal.pone.0075690 (PMC3783415; doi:10.1371/journal.pone.0075690)
Supplement: Table S4 — Cosinor analysis of liver expression profiles on RF. (DOCX) [file pone.0075690.s007.docx]

Table S4. Cosinor analysis of liver expression profiles on RF.

| **Gene** | **Per1** | | **Per2** | | **Rev-erbα** | | **Bmal1** | | **Bmal2** | | **Wee1** | | **Dbp** | | **E4bp4** | |
| --- | --- | --- | --- | --- | --- | --- | --- | --- | --- | --- | --- | --- | --- | --- | --- | --- |
| **Strain** | **SHR** | **Wistar** | **SHR** | **Wistar** | **SHR** | **Wistar** | **SHR** | **Wistar** | **SHR** | **Wistar** | **SHR** | **Wistar** | **SHR** | **Wistar** | **SHR** | **Wistar** |
| **P** | 0.0016 | 0.0502 | 0.0002 | < .0001 | < .0001 | < 0.001 | < 0.001 | < 0.001 | 0.0005 | 0.0015 | < .0001 | < .0001 | 0.0021 | < .0001 | < .0001 | 0.0293 |
| **R^2^** | 0.5095 | 0.1706 | 0.6304 | 0.6566 | 0.8546 | 0.6729 | 0.9417 | 0.8338 | 0.5733 | 0.3334 | 0.7201 | 0.7933 | 0.4973 | 0.5677 | 0.7905 | 0.1979 |
| **Mesor** | 0.0998 | 0.1564 | 0.3412 | 0.2643 | 0.4234 | 0.5483 | 0.2760 | 0.2717 | 5.9210 | 4.3150 | 0.1492 | 0.1405 | 0.3787 | 0.6120 | 1.5830 | 1.8370 |
| **SE** | 0.0108 | 0.0155 | 0.0175 | 0.0095 | 0.0419 | 0.0604 | 0.0141 | 0.0169 | 0.2141 | 0.3067 | 0.0136 | 0.0058 | 0.0711 | 0.0691 | 0.0681 | 0.1125 |
| **Amp** | 0.0661 | 0.0550 | 0.1428 | 0.1111 | 0.5736 | 0.6619 | 0.3347 | 0.3103 | 1.4730 | 1.7500 | 0.1392 | 0.0928 | 0.4112 | 0.6646 | 0.7437 | 0.4277 |
| **SE** | 0.0154 | 0.0216 | 0.0266 | 0.0142 | 0.0559 | 0.0820 | 0.0198 | 0.0246 | 0.3020 | 0.4407 | 0.0205 | 0.0084 | 0.0981 | 0.1027 | 0.0903 | 0.1530 |
| **Acro** | 3.34 | 9.34 | 5.90 | 5.79 | 23.17 | 25.69 | 14.82 | 16.07 | 14.91 | 20.50 | 6.18 | 8.12 | 2.24 | 4.96 | 12.39 | 13.75 |
| **SE** | 1.16 | 3.01 | 0.67 | 0.28 | 0.71 | 0.73 | 0.38 | 0.50 | 1.01 | 1.25 | 0.76 | 0.30 | 1.14 | 1.00 | 0.47 | 0.80 |

| **Gene** | **Nampt** | | **Ppara** | | **Pparg** | | **Pgc1α** | | **Prkab2** | | **Hdac3** | | **Hif1a** | | **Ppp1r3c** | |
| --- | --- | --- | --- | --- | --- | --- | --- | --- | --- | --- | --- | --- | --- | --- | --- | --- |
| **Strain** | **SHR** | **Wistar** | **SHR** | **Wistar** | **SHR** | **Wistar** | **SHR** | **Wistar** | **SHR** | **Wistar** | **SHR** | **Wistar** | **SHR** | **Wistar** | **SHR** | **Wistar** |
| **P** | < .0001 | < .0001 | 0.0185 | 0.9462 | 0.5292 | 0.3026 | 0.9290 | 0.4234 | 0.0378 | 0.3763 | 0.9593 | 0.8906 | 0.6786 | 0.6449 | 0.0886 | 0.0038 |
| **R^2^** | 0.7318 | 0.6774 | 0.3580 | 0.0035 | 0.0683 | 0.0720 | 0.0082 | 0.0523 | 0.3051 | 0.0611 | 0.0046 | 0.0072 | 0.0422 | 0.0271 | 0.2361 | 0.2937 |
| **Mesor** | 0.2967 | 0.3671 | 0.5592 |  |  |  |  |  | 1.0300 |  |  |  |  |  |  | 0.7201 |
| **SE** | 0.0121 | 0.0163 | 0.0398 |  |  |  |  |  | 0.1050 |  |  |  |  |  |  | 0.0514 |
| **Amp** | 0.1269 | 0.1931 | 0.1821 |  |  |  |  |  | 0.3915 |  |  |  |  |  |  | 0.2810 |
| **SE** | 0.0181 | 0.0237 | 0.0578 |  |  |  |  |  | 0.1393 |  |  |  |  |  |  | 0.0771 |
| **Acro** | 6.55 | 8.01 | 3.88 |  |  |  |  |  | 12.51 |  |  |  |  |  |  | 18.35 |
| **SE** | 0.46 | 0.60 | 1.30 |  |  |  |  |  | 1.54 |  |  |  |  |  |  | 0.84 |

Acro (acrophase); Amp (amplitude); R^2^ (coefficient of determination); P (*p*-value for cosinor model)
