# Supplementary material for: Increased Sensitivity of the Circadian System to Temporal Changes in the Feeding Regime of Spontaneously Hypertensive Rats - A Potential Role for Bmal2 in the Liver
Source: PLoS One. 2013 Sep 25;8(9):e75690. doi: 10.1371/journal.pone.0075690 (PMC3783415; doi:10.1371/journal.pone.0075690)
Supplement: Table S5 — Cosinor analysis of colon expression profiles on RF. (DOCX) [file pone.0075690.s008.docx]

Table S5. Cosinor analysis of colon expression profiles on RF.

| **Gene** | **Per1** | | **Per2** | | **Rev-erbα** | | **Bmal1** | | **Bmal2** | | **Wee1** | | **Dbp** | | **E4bp4** | |
| --- | --- | --- | --- | --- | --- | --- | --- | --- | --- | --- | --- | --- | --- | --- | --- | --- |
| **Strain** | **SHR** | **Wistar** | **SHR** | **Wistar** | **SHR** | **Wistar** | **SHR** | **Wistar** | **SHR** | **Wistar** | **SHR** | **Wistar** | **SHR** | **Wistar** | **SHR** | **Wistar** |
| **P** | 0.0014 | 0.2336 | < .0001 | 0.0002 | < .0001 | < .0001 | < .0001 | < .0001 | 0.0722 | 0.8248 | 0.0016 | 0.0001 | < .0001 | 0.0002 | 0.6077 | 0.3694 |
| **R^2^** | 0.5191 | 0.0869 | 0.8600 | 0.4177 | 0.8220 | 0.4780 | 0.7028 | 0.6954 | 0.2532 | 0.0124 | 0.5104 | 0.4336 | 0.7289 | 0.4179 | 0.0538 | 0.0603 |
| **Mesor** | 0.1038 |  | 0.4688 | 0.8407 | 0.1745 | 0.3763 | 0.5548 | 0.6466 |  |  | 0.2396 | 0.3065 | 1.1960 | 2.1690 |  |  |
| **SE** | 0.0110 |  | 0.0212 | 0.0426 | 0.0130 | 0.0330 | 0.0334 | 0.0437 |  |  | 0.0159 | 0.0144 | 0.1154 | 0.2097 |  |  |
| **Amp** | 0.0675 |  | 0.3183 | 0.2745 | 0.1557 | 0.2461 | 0.3115 | 0.5586 |  |  | 0.1025 | 0.0960 | 1.1140 | 1.4010 |  |  |
| **SE** | 0.0154 |  | 0.0312 | 0.0584 | 0.0176 | 0.0458 | 0.0481 | 0.0654 |  |  | 0.0237 | 0.0198 | 0.1614 | 0.2987 |  |  |
| **Acro** | 2.87 |  | 6.77 | 10.18 | 0.24 | 2.39 | 15.55 | 17.50 |  |  | 6.90 | 10.12 | 2.70 | 3.16 |  |  |
| **SE** | 1.36 |  | 0.30 | 1.34 | 0.47 | 1.10 | 0.70 | 0.41 |  |  | 0.87 | 0.44 | 0.53 | 1.06 |  |  |

| **Gene** | **Nampt** | | **Ppara** | | **Pparg** | | **Pgc1α** | | **Prkab2** | | **Hdac3** | | **Hif1a** | | **Ppp1r3c** | |
| --- | --- | --- | --- | --- | --- | --- | --- | --- | --- | --- | --- | --- | --- | --- | --- | --- |
| **Strain** | **SHR** | **Wistar** | **SHR** | **Wistar** | **SHR** | **Wistar** | **SHR** | **Wistar** | **SHR** | **Wistar** | **SHR** | **Wistar** | **SHR** | **Wistar** | **SHR** | **Wistar** |
| **P** | 0.0002 | < .0001 | 0.8970 | 0.8589 | < .0001 | 0.0049 | 0.4293 | 0.4220 | 0.7310 | 0.0436 | 0.8622 | 0.2823 | 0.3965 | 0.0238 | 0.2875 | 0.1396 |
| **R^2^** | 0.6373 | 0.5545 | 0.0120 | 0.0095 | 0.7205 | 0.2831 | 0.0897 | 0.0525 | 0.0342 | 0.1830 | 0.0163 | 0.0760 | 0.0977 | 0.2083 | 0.1293 | 0.1158 |
| **Mesor** | 0.1333 | 0.2327 |  |  | 0.8353 | 1.2300 |  |  |  | 1.3000 |  |  |  | 3.0900 |  |  |
| **SE** | 0.0046 | 0.0088 |  |  | 0.0366 | 0.0663 |  |  |  | 0.1177 |  |  |  | 0.1961 |  |  |
| **Amp** | 0.0369 | 0.0820 |  |  | 0.3704 | 0.3287 |  |  |  | 0.4088 |  |  |  | 0.8149 |  |  |
| **SE** | 0.0067 | 0.0130 |  |  | 0.0545 | 0.0932 |  |  |  | 0.1555 |  |  |  | 0.2828 |  |  |
| **Acro** | 6.23 | 7.39 |  |  | 5.05 | 9.19 |  |  |  | 12.49 |  |  |  | 15.62 |  |  |
| **SE** | 0.68 | 0.66 |  |  | 0.48 | 1.31 |  |  |  | 1.65 |  |  |  | 1.50 |  |  |

Acro (acrophase); Amp (amplitude); R^2^ (coefficient of determination); P (*p*-value for cosinor model)
